# Advanced Drug Delivery in Pediatric Neurology: Developmental Barriers, Nanocarrier Strategies, and Translational Challenges

**DOI:** 10.3390/ijms27198727

**Published:** 2026-09-29

**Authors:** Raluca Ioana Teleanu, Andreea Gabriela Bratu, Adelina-Gabriela Niculescu, Radu Ștefan Perjoc, Eugenia Roza, Daniel Mihai Teleanu, Oana Aurelia Vladâcenco, Ana Cojocaru, Alexandru Mihai Grumezescu, Iustina Mihaela Pintilie

**Affiliations:** 1 “Carol Davila” University of Medicine and Pharmacy, 020021 Bucharest, Romania; raluca.teleanu@umfcd.ro (R.I.T.); eugenia.roza@umfcd.ro (E.R.); daniel.teleanu@umfcd.ro (D.M.T.); oana-aurelia.vladacenco@drd.umfcd.ro (O.A.V.); ana.damaschin@rez.umfcd.ro (A.C.); iustina-mihaela.pintilie@rez.umfcd.ro (I.M.P.); 2 Faculty of Chemical Engineering and Biotechnologies, National University of Science and Technology POLITEHNICA Bucharest, 011061 Bucharest, Romania; andreea.bratu2910@stud.fim.upb.ro (A.G.B.); adelina.niculescu@upb.ro (A.-G.N.); agrumezescu@upb.ro (A.M.G.); 3 Research Institute of the University of Bucharest—ICUB, University of Bucharest, 050657 Bucharest, Romania

**Keywords:** pediatric neurology, drug delivery, targeted nanocarriers, blood–brain barrier, epilepsy, brain tumor, pediatric clinical research

## Abstract

Treating neurological disorders in children is particularly challenging due to the dynamic maturation of the developing brain, age-dependent pharmacokinetics, and evolving structural and functional properties of the blood–brain barrier (BBB). This review explores the latest advances in drug-delivery systems tailored for pediatric neurology, emphasizing strategies to address physiological and developmental determinants of treatment response across infancy, childhood, and adolescence. The paper begins by outlining the distinct challenges of drug delivery in children, focusing on BBB maturation, transporter expression, metabolic capacity, and neurodevelopmental vulnerability. Emerging delivery systems, such as liposomes, polymeric nanoparticles, dendrimers, micelles, gold nanoparticles, and gene- and RNA-based vectors, are further reviewed in relation to major pediatric neurological conditions. These platforms demonstrate promise in enhancing drug bioavailability, targeting specific brain regions, and minimizing systemic toxicity. This paper also covers important safety, regulatory, and ethical considerations in pediatric populations, with a focus on the need for age-appropriate design and thorough clinical validation. Concluding with emerging trends and future directions, this work serves as a comprehensive resource for advancing pediatric neurological therapeutics through state-of-the-art drug delivery technologies.

## 1. Introduction

### 1.1. Brief General Background on Pediatric Neurology

Neurological conditions have risen to become the primary source of health decline, significantly affecting individuals’ overall health and quality of life, ranking among the primary contributors to global mortality and disability [1,2]. Neurological disorders in children constitute a considerable segment of the global disease burden, as they lead to increased rates of premature death and years lived with disabilities. Between 1990 and 2015, there has been a notable decline in this burden, particularly reflected in reduced mortality rates; however, the number of years lived with disabilities has seen only a slight increase. Despite these advancements, certain areas—especially South Asia and sub-Saharan Africa—continue to experience high levels of burden due to elevated occurrences of prematurity, neonatal encephalopathy, and central nervous system (CNS) infections [3]. A global burden assessment for 37 neurological disorders was conducted within the latest Global Burden of Disease 2021 framework, which includes neurodevelopmental disorders, neurodegenerative diseases, neuromuscular conditions, cerebrovascular disorders, CNS infections, and neurological sequelae of systemic diseases. For children under the age of five, the primary contributors to disability-adjusted life years (DALYs) worldwide included neonatal encephalopathy, meningitis, and neural tube defects. In contrast, for individuals aged 5 to 19 years, the top three causes of DALYs were migraines, neurological issues stemming from preterm birth, and epilepsy [2].

**Figure 1:**
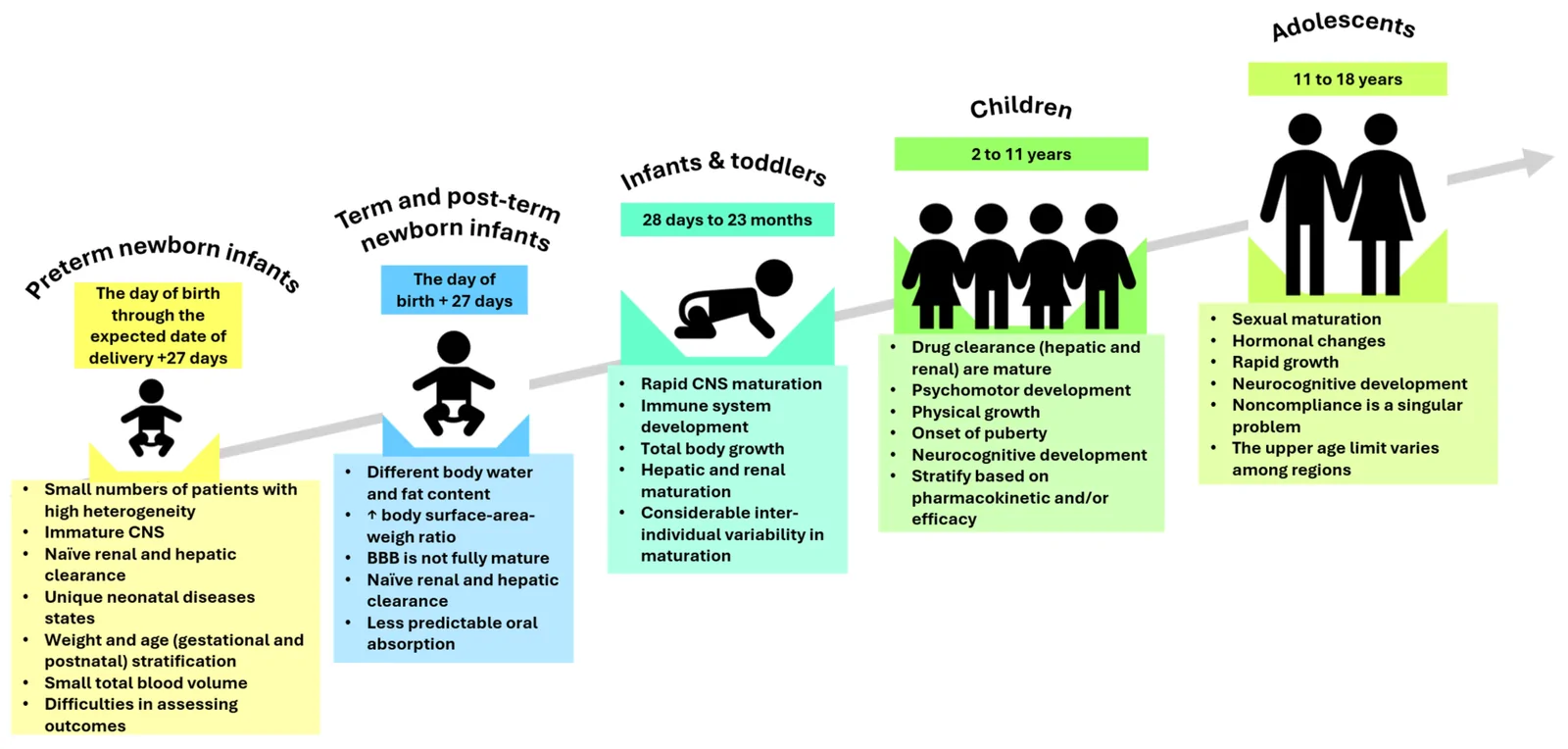
Classification of pediatric patients by age according to the International Council for Harmonization (ICH) guideline E11 (R1). It is assumed that there is significant variation in the classification of developmental stages (physical, cognitive, and psychosocial) across children of different ages. BBB—blood–brain barrier; CNS—central nervous system; ↑—rise. Adapted from [4].

According to the International Council for Harmonization (ICH) topic E11 (CPMP/ICH/2711/99) and ICH E11 (R1), the pediatric population can be divided into the following subgroups: preterm newborn infants, term along with post-term newborn infants, infants and toddlers, children, and adolescents, as shown in Figure 1 [4,5].

The complex field of pediatric pharmacotherapy necessitates close attention to age-specific differences in medication response. Physiological variations between newborns and teenagers substantially influence medication pharmacokinetics and pharmacodynamics, requiring specialized treatment strategies. For example, hormonal fluctuations in teenagers may influence pharmacokinetic profiles, whereas newborns’ underdeveloped hepatic enzyme systems impact drug metabolism. These variations underscore the importance of using age-appropriate medication formulations and dosing regimens to optimize therapeutic outcomes [6]. Due to logistical and ethical constraints that frequently prevent thorough data collection, the pediatric population also faces difficulties because of the scarcity of large-scale clinical studies that include children. Hence, pediatric medication development now heavily relies on extrapolated adult data, which may not always adequately reflect the needs of younger patients. Therefore, to close these gaps and enhance the safety and effectiveness of pediatric pharmacotherapy, developments in personalized medicine and physiologically based pharmacokinetic modeling are essential [7,8].

In pediatric neurology, the limited number of approved drugs often results in off-label use, where medications authorized for adults are prescribed to children without formal pediatric approval. This practice is not always appropriate, given the significant physiological differences between children and adults (e.g., drug metabolism, absorption, CNS development, and long-term effects) that affect treatment efficacy and safety. As a result, disease management and treatments that are effective in adults may have different outcomes in pediatric populations. Despite these challenges, clinicians may rely on FDA drug programs and informed clinical judgment to guide off-label prescribing when potential benefits for children are supported by available evidence [9,10].

### 1.2. Brain Physiology from Preterm to Adult

A clear understanding of brain physiology and developmental barrier maturation is essential to contextualize the therapeutic challenges discussed in this review.

Although the primary arterial pattern of the cerebral circulation is established before birth, the brain’s vascular network is not fully mature at birth. Postnatally, the cerebral microvasculature undergoes continued angiogenesis, pruning, endothelial specialization, and functional maturation of neurovascular coupling, which extends through infancy and early childhood [11,12,13].

The blood–brain barrier (BBB) phenotype of brain capillaries is characterized by highly specialized endothelial cells joined by continuous tight junctions (TJs) that restrict paracellular permeability, minimal vesicular (transcytotic) transport, and a rich repertoire of selective transporters and metabolic enzymes. This unique phenotype distinguishes brain microvessels from peripheral capillaries and ensures controlled molecular exchange between the blood and neural tissue. In addition, pericytes and astrocytic endfeet closely envelop the endothelial layer, contributing to barrier induction, maintenance, and regulation [14,15]. In addition to the BBB, the blood–cerebrospinal fluid barrier (BCSFB) and other specialized CNS interfaces contribute to regulated molecular exchange [16].

The BBB is a significant physiological impediment to the management of neurological conditions, particularly in children, whose brains are not fully developed at birth and take many years to mature. Although key barrier properties are already established at birth, BBB transport, metabolic, and regulatory functions continue to undergo postnatal maturation. The heterogeneity of barrier modifications reported within an individual tumor and among different pediatric tumor types constitutes an additional hurdle [17,18,19]. BBB integrity is affected by the tumor type, molecular subtype, and anatomical location, and it differs considerably between pediatric and adult brain tumors. The distinctive developmental stage of the pediatric BBB may further limit the penetration of therapeutic drugs, particularly large molecules such as monoclonal antibodies (mAbs), thereby impacting treatment outcomes [20,21].

### 1.3. Drug Delivery to the Brain

Given these biological constraints, over the years, several approaches have been developed to enhance drug delivery to the brain by circumventing the BBB.

In medulloblastoma, the BBB phenotype differs across molecular subtypes. WNT-medulloblastoma lacks a functional BBB, exhibiting a profound disruption characterized by decreased endothelial TJ proteins (claudin-5, occludin, and ZO-1), loss of basement membrane organization, and increased paracellular permeability. These tumors also exhibit enhanced transcytosis and reduced expression of efflux transporters, such as P-gp, allowing chemotherapeutic agents to more readily penetrate the tumor vasculature [22,23,24]. On the other hand, the SHH subtype has an intact BBB, thereby affecting the bioavailability of systemic therapies. Herein, endothelial junctional proteins are preserved, P-gp and BCRP efflux transporters remain highly expressed, and transcytosis rates are low. As a result, standard systemic therapies show markedly reduced intratumoral drug accumulation in SHH tumors despite therapeutic plasma levels [22,24]. Passive targeting via the EPR-like effect should be considered constrained and inconsistent in brain tumors, including WNT-medulloblastoma, despite their comparatively more permeable vasculature. Data indicate that BBB rupture in CNS tumors is diverse and spatially heterogeneous, leading to uneven nanoparticle accumulation via passive processes. In this context, active targeting techniques are more suitable, especially those that utilize receptor-mediated transcytosis pathways across the BBB. Ligand-functionalized nanoparticles that target transferrin receptors or low-density lipoprotein receptors can promote transport across endothelial cells and increase tumor accumulation [25,26].

The permeability, bioavailability, and clinical response of systemically delivered treatment drugs are all affected by the variation in BBB disruption that exists between adult and pediatric brain tumors. Pediatric tumors often arise in a developmentally immature vasculature where angiogenesis is ongoing, endothelial fenestrations are limited, and transporter expression patterns are still evolving. Adult tumors, particularly high-grade gliomas, typically exhibit more extensive vascular remodeling, hypoxia-driven VEGF signaling, and basement membrane degradation, leading to a more heterogeneous but generally leakier BBB. Consequently, drug permeability, distribution, and response may be greater in adult high-grade gliomas than in pediatric medulloblastomas, where barrier tightness remains a major therapeutic obstacle [20,27].

Consequently, attention has progressively shifted to non-invasive methods to improve the transport of therapeutic drugs into the brain while preserving the integrity of the BBB [28]. The efficacy of targeted therapy has been clearly shown for a particular group of pediatric brain tumors. Over the past decade, understanding the molecular biology of pediatric brain tumors has advanced with the development of genomic platforms. Nonetheless, despite evident response rates, issues regarding adequate BBB penetration, effective dose, treatment duration, mechanisms of resistance, and potential combination strategies to address these challenges must be examined in forthcoming studies. In this context, future trials need to establish suitable endpoints for evaluating therapeutic responses and both short-term and long-term toxicity in the maturing and developing child [29,30].

### 1.4. Review Aim and Search Methodology

This narrative review addresses major challenges in pediatric CNS drug delivery, such as the developmentally evolving BBB, age-dependent pharmacokinetics, and systemic toxicity, to examine novel approaches, including nanocarriers, stimuli-responsive systems, and non-invasive delivery methods. These advanced technologies seek to enhance targeting, minimize adverse effects, and facilitate personalized, age-appropriate therapies. This paper subsequently examines their therapeutic applications, encompassing epilepsy, brain tumors, and neurodegenerative disorders. The review further considers the safety and translational challenges associated with these technologies, with emphasis on the development of age-appropriate therapeutic strategies.

In this context, a structured literature search was conducted in PubMed, Web of Science, and Google Scholar, primarily considering English-language publications available through August 2026. Search terms were used in different combinations and included “pediatric neurology”, “pediatric drug delivery”, “blood–brain barrier”, “nanocarriers”, “nanoparticles”, “liposomes”, “polymeric nanoparticles”, “dendrimers”, “micelles”, “gene delivery”, “RNA delivery”, “brain tumors”, “epilepsy”, and “neurodegenerative disorders”. Studies were selected based on their relevance to developmental barriers, CNS drug-delivery strategies, pharmacokinetics, biodistribution, efficacy, safety, and clinical translation.

The disease areas discussed in Section 4 were selected as representative applications of advanced CNS drug-delivery strategies. Brain tumors, epilepsy, and selected neurodegenerative disorders were included because they provide sufficiently developed preclinical, translational, or clinical evidence to illustrate distinct delivery challenges and technological approaches, including BBB-targeted nanocarriers, controlled and stimuli-responsive systems, local delivery, and gene- or RNA-based strategies.

Given the limited and heterogeneous nature of the pediatric literature on advanced drug delivery systems for the CNS, this analysis distinguishes between evidence obtained directly from pediatric populations or relevant models for development and extrapolated data from adult humans, adult animal models, and in vitro systems. Primary studies have been prioritized for statements on transport mechanisms, pharmacokinetics, biodistribution, efficacy, and safety, while secondary sources have been selectively retained for general context and domains where the pediatric primary literature remains insufficient. Where data are missing for specific pediatric age groups, this is explicitly indicated as a limitation of current evidence and a priority for future research. Publications not available in English and studies outside the scope of pediatric CNS drug delivery were excluded.

## 2. Drug Delivery Challenges in Pediatric Neurology

The BBB represents a dynamic neurovascular interface whose permeability and transport properties are regulated by modifications in TJ proteins, transporter expression, and endothelial cell function [14,15]. These mechanisms allow the BBB to adapt to physiological and pathological changes, including during development, without the barrier normally facing rapid, nonspecific variations in permeability. Pericytes, brain blood flow, and neurovascular regulation can further influence the contribution and distribution of substances at the brain level [31,32,33]. In the pediatric context, the dynamic and developmentally dependent nature of these processes has direct implications for drug exposure in the brain and for the design of CNS-dedicated delivery systems.

### 2.1. Developmental Variations in the Pediatric Blood–Brain Barrier

Human brain development is a highly coordinated process that involves both the formation of the CNS barrier and the maturation of parenchymal cells. It begins at gestational week 3 (GW3) with the differentiation of ectodermal neuroepithelial cells into the neural plate and neural tube, initiating CNS development. By GW5, vascularization separates the CNS from the periphery; the choroid plexus forms around GW7, and most meningeal structures are established by GW12 [34,35,36]. TJ proteins (e.g., occludin and claudin-5), detected in the fetal brain by GW 16, provide an effective barrier against albumin infiltration, with adult-like BBB staining patterns evident by GW18 [37,38]. Pyramidal neurons originate from proliferating progenitor cells, including radial glial cells, as early as GW 5. Neurogenesis, persisting until early postnatal life, peaks between GW 9 and 13, with neurons migrating radially through the subventricular zone, while synaptogenesis begins at GW8 and is refined by microglia- and astrocyte-mediated pruning [35]. Microglia appear before vascular sprouting (GW 5), astrocytes emerge by GW 15, and oligodendrocyte progenitor cells by GW 17. The BBB then develops further through interaction with pericytes (PCs) and astrocytes inside the neurovascular unit (NVU) [39].

After birth, the BBB continues to mature on both structural and functional levels. TJ proteins increase in abundance and organization throughout the early postnatal period, progressively lowering paracellular permeability. Astrocytes become fully integrated into the NVU within the first weeks after birth, and their endfeet coverage of microvessels improves barrier polarity and transporter localization. PC density and endothelial–pericyte signaling also increase postnatally, further strengthening vessel stability and barrier tightness [14,15].

Efflux transporters, such as P-gp (ABCB1) and BCRP (ABCG2), exhibit postnatal upregulation and reach adult-like expression during infancy and childhood, thereby enhancing the metabolic and pharmacological barrier [40]. In parallel, nutrient transport systems such as glucose transporter 1 (GLUT1) mature and expand to meet the metabolic needs of the developing brain [41]. Microglial and peripheral immune cell interactions with the BBB become increasingly tightly regulated throughout childhood and adolescence, contributing to immune surveillance while maintaining immune privilege [42,43].

These processes continue throughout adolescence along with brain myelination, synaptic pruning, and vascular remodeling. Although many BBB features are established in early life, complete maturation of transport, metabolic, and immune barrier functions occurs progressively and corresponds to the evolving physiological needs of the developing CNS [17,43].

**Figure 2:**
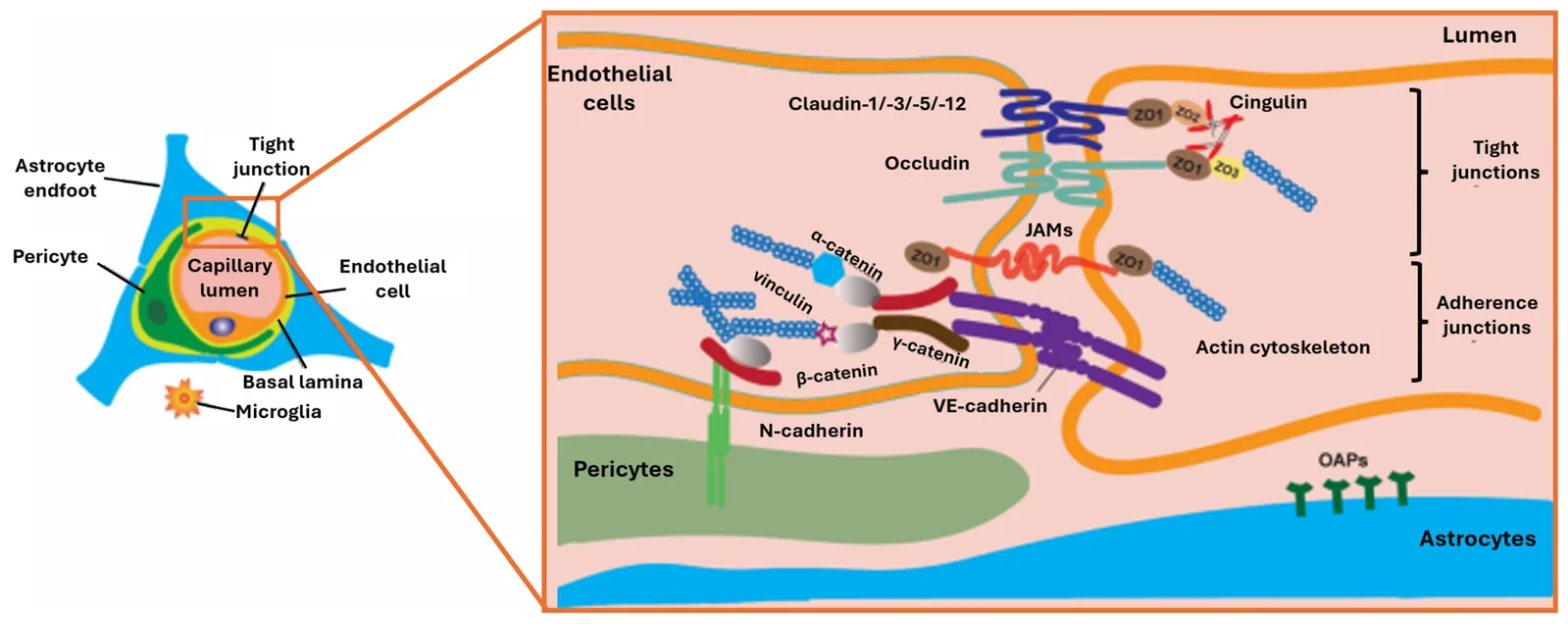
Molecular organization and cell interaction within the neurovascular unit. Reprinted from [54].

The NVU consists of microvascular brain endothelial cells, the basement membrane, pericytes, perivascular astrocyte extensions, microglia, and neural elements that together maintain BBB integrity and transport function [44] (Figure 2). Brain endothelial cells differentiate from peripheral endothelium through the presence of continuous interendothelial junctions, reduced nonspecific transcytosis, and specialized transport mechanisms mediated by transporters, receptors, or adsorption that strictly control the entry of molecules into the CNS [45,46]. Pericytes and astrocyte extensions further support endothelial maturation, vascular stability, barrier polarization, and regulation of brain blood flow [31,47,48]. At a molecular level, paracellular permeability is mainly controlled by TJ proteins, including claudin-5, occludin, and zonula occludens proteins, whose expression and organization contribute to maintaining BBB integrity [49,50,51,52,53].

In addition, endothelial proteins involved in transcellular permeability and vascular remodeling, including PV1 and the VEGF/VEGFR-2 signaling pathway, become significantly relevant in developmental or pathological conditions in which BBB function is altered [55,56,57,58]. Experimental evidence indicates that aggression during early developmental periods can lead to persistent modifications in BBB properties; however, these observations should not be interpreted as indicating a generally increased pediatric physiological BBB permeability. In a neonatal model of induced excitotoxicity through monosodium glutamate administration to rats, Fajardo-Fregoso and collaborators [59] have identified persistent modifications of the proteins associated with this barrier at adult age, including the reduction in claudin-5 and ZO-1 expression in certain brain regions, the increase in PV1 and VEGFR-2 expression, and an enhanced susceptibility to hypertonic stress-induced permeabilization; VEGFR-2 inhibition has reduced this vulnerability. Hence, the study demonstrates that neonatal excitotoxic aggression can remodel BBB function in the long term, but it does not provide evidence for generally increased BBB permeability in healthy children.

Data obtained directly from human brain tissue also support a more nuanced interpretation of BBB maturation. P-glycoprotein (P-gp/ABCB1) is detectable in brain microvascular endothelium from the fetal period onward, with its expression modified during development [40,60]. Analysis of brain tissue from fetuses, newborns, children, and adults demonstrates that ABC transporter maturation is dependent on both age and transporter type; the expression of P-gp and BCRP at the cortical BBB increases with age, while other transporters exhibit different ontogenetic models [61]. Moreover, human fetal brain endothelial cells isolated from early- and mid-gestation express functional P-gp and BCRP transporters [62]. Consequently, the developing BBB must be considered a functional interface with age- and transporter-dependent properties, rather than simply an “immature” or more uniformly permeable barrier.

The transport barrier includes several efflux transporters, including P-glycoprotein (P-gp), breast cancer resistance protein (BCRP), and OATP. These efflux systems may have overlapping substrate affinities, such as P-gp and BCRP, which actively extrude substances, including xenobiotics, from ECs into the bloodstream, therefore diminishing CNS exposure [63,64]. Finally, the presence of drug-metabolizing enzymes in brain ECs, including cytochrome P450 (CYP) enzymes (CYP1B1, CYP2U1, and CYP3A4), establishes a metabolic barrier [65]. CYP mRNAs have been identified in several regions of the human brain, contributing to the pathogenesis of neurodegenerative diseases by metabolizing drugs and fatty acids. CYP2U1 metabolizes fatty acids such as arachidonic acid, whereas CYP1B1 is involved in the metabolism of endogenous substances inside the CNS. CYP3A4 was also shown to be functionally expressed in the drug-resistant epileptic BBB and to oxidize a broad array of xenobiotics, including antiepileptic medications [65,66].

### 2.2. Age-Dependent Pharmacokinetics and Limitations of Conventional Delivery Systems

Pharmacokinetics and/or pharmacodynamics may also differ across pediatric patients of varying ages. Nonetheless, the most significant discrepancies related to adult pharmacokinetic profiles are evident in infants under 2 years of age. The development of the liver and kidneys, along with changes in body composition, are considered the primary contributors to pharmacokinetic variability. Therefore, in addition to pharmacodynamic characteristics, understanding age-related alterations in drug distribution, absorption, and elimination is essential to enhance pharmacological effectiveness and prevent toxicity [67,68,69].

Age-dependent changes in absorption also contribute to pharmacokinetic variability in the pediatric population. Progressive maturation of gastrointestinal pH and motility, intestinal metabolism, and transporters, as well as differences between cutaneous and rectal absorption, can modify drug bioavailability, especially in newborns and infants [68,69,70,71]. These features limit direct extrapolation of adult formulations and dosing regimens, supporting the need to develop age-tailored administration strategies [68,72,73].

Intrathecal (IT) and intracerebral injections in pediatric care are invasive procedures that are often employed for treating severe neurological conditions, including cerebral palsy, spasticity, dystonia, and other movement abnormalities. These were well tolerated in pediatric patients, with the most commonly reported adverse effects post-treatment generally brief, moderate, and self-limiting. This may be attributed to the small amount of toxins anticipated to reach systemic circulation [74,75]. However, the retrospective design, limited sample size, and single-center context of existing studies constrain the robustness and generalizability of the results and may create bias. IT therapies in pediatric neurology involve the direct administration of a drug into the cerebrospinal fluid, thereby bypassing the BBB for targeted treatment. Principal therapies encompass intrathecal baclofen (ITB) for severe spasms, methotrexate (MTX) for acute lymphoblastic leukemia and medulloblastoma, nusinersen for spinal muscular atrophy (SMA), and experimental treatments such as corticosteroids or stem cells for neuroinflammation [76,77,78].

An essential benefit of ITB is its efficacy in diminishing muscle stiffness, cramping, and pain while enhancing functionality and quality of life. The primary advantage is personalized dosing, which enables clinicians to improve outcomes while reducing side effects. However, risks associated with the pump/catheter include infection, dislocation, or mechanical failure necessitating revision [79,80]. IT delivery of antisense oligonucleotides has transformed the management of spinal muscular atrophy (SMA type 1), with agents like nusinersen demonstrating meaningful improvements in motor outcomes and survival. Nusinersen treatment initiated before 12 months of age, particularly before 3 months, produced the most favorable motor outcomes in children with SMA type 1. Early onset correlates with enhanced motor milestones and a lower need for ventilatory support. Additional information on the drug’s efficacy and safety requires larger sample sizes and longer follow-up periods to yield more reliable results [81,82,83]. IT corticosteroids have garnered heightened interest in recent years for the management of diverse neuroimmunological conditions, encompassing those primarily involving innate and adaptive immune mechanisms. IT corticosteroid injections may reduce systemic exposure and thereby mitigate certain adverse effects. It is crucial to assess corticosteroid formulations for their appropriateness for IT injection, as some excipients and additives may exhibit neurotoxicity [84]. IT methotrexate (MTX) showed beneficial therapeutic effects in children with high-risk medulloblastoma, especially in controlling metastatic disease. IT administration allows direct delivery into CSF, improving drug exposure in the CNS [78].

Clinically proven and newly developed CNS-targeted delivery techniques might be beneficial when discussing pediatric neurogenetic diseases. Intracerebroventricular enzyme replacement therapy has emerged as a validated treatment for neuronal ceroid lipofuscinosis type 2 (CLN2), enabling direct delivery into the ventricular system to circumvent the BBB and achieve therapeutic concentrations in the brain. Given the effects of enzyme replacement therapy on domains beyond motor and language abilities (e.g., quality of life, movement-related conditions, seizures, behavior), future CLN2 rating systems should encompass these domains [85,86].

Novel strategies, such as IT gene therapy, underscore the evolving therapeutic landscape for rare neurogenetic disorders, including giant axonal neuropathy. This method seeks to facilitate extensive neuronal transduction by administering viral vectors directly into the cerebrospinal fluid, thereby reducing systemic exposure [87].

A clinically evaluated example of formulation-based drug delivery in pediatric neurology is intra-erythrocyte dexamethasone sodium phosphate for ataxia telangiectasia. The multicenter phase 3 ATTeST trial evaluated this erythrocyte-based approach directly in children, providing pediatric clinical evidence for a strategy designed to modify corticosteroid exposure through an alternative delivery platform [84]. Its inclusion is particularly relevant to this review because it illustrates how modification of drug disposition, rather than introduction of a new active compound, can be investigated as a means of optimizing therapy in a pediatric neurodegenerative disorder [88].

General anesthesia and/or sedation are commonly administered to children to alleviate procedure-related anxiety, discomfort, and distress, hence enhancing the general efficacy rate of lumbar puncture. Nevertheless, escalating apprehensions regarding the effects of recurrent anesthesia administration on the developing neural systems of newborns and young children must be meticulously evaluated before scheduling repeated treatments [74,75].

The compromised BBB neuroprotective characteristics are a significant obstacle to medication administration to the brain, particularly in neurological diseases characterized by hyperactive efflux transporters. Consequently, medications may fail to reach target tissues or attain enough concentrations in the brain, a difficulty seen in several CNS illnesses to date. Drug delivery has emerged as a significant area of study in the medical and pharmaceutical sectors during the last two decades. Recent investigations have demonstrated novel techniques for bypassing the BBB for medication delivery [89,90,91]. Local delivery methods, such as convection-enhanced delivery (CED) and biodegradable polymer-based drug release, circumvent the BBB by administering treatments directly to the desired location. Strategies for penetrating the BBB, including nanoparticles (NPs), exosomes, and vectors, exploit the brain’s intrinsic transport mechanisms. Strategies that disrupt the BBB, such as focused ultrasound (FUS), temporarily augment the barrier’s permeability to improve medication biodistribution. Intranasal administration provides a non-invasive and direct pathway, whereas receptor-mediated transport utilizes pre-existing transport pathways [92,93,94,95].

## 3. Novel Drug Delivery Systems for Pediatric Neurology

CNS drug delivery is limited by reduced brain exposure, nonspecific distribution, systemic clearance, and efflux mechanisms at the BBB, all of which can diminish therapeutic concentrations in brain tissue [96,97,98,99,100,101,102,103,104]. Even though local or invasive methods can partially overcome these barriers, their repeated use is limited by procedural risks and clinical applicability, especially in the pediatric population.

In this context, nanoparticulate systems protect and deliver the therapeutic load, improve drug solubility and stability, prolong circulation, and enable controlled or targeted release [97,105,106]. For pediatric utilization, the evaluation of these platforms must include age-dependent pharmacokinetics, biocompatibility, dosing flexibility, and potential long-term effects on the developing brain [21,107,108].

**Figure 3:**
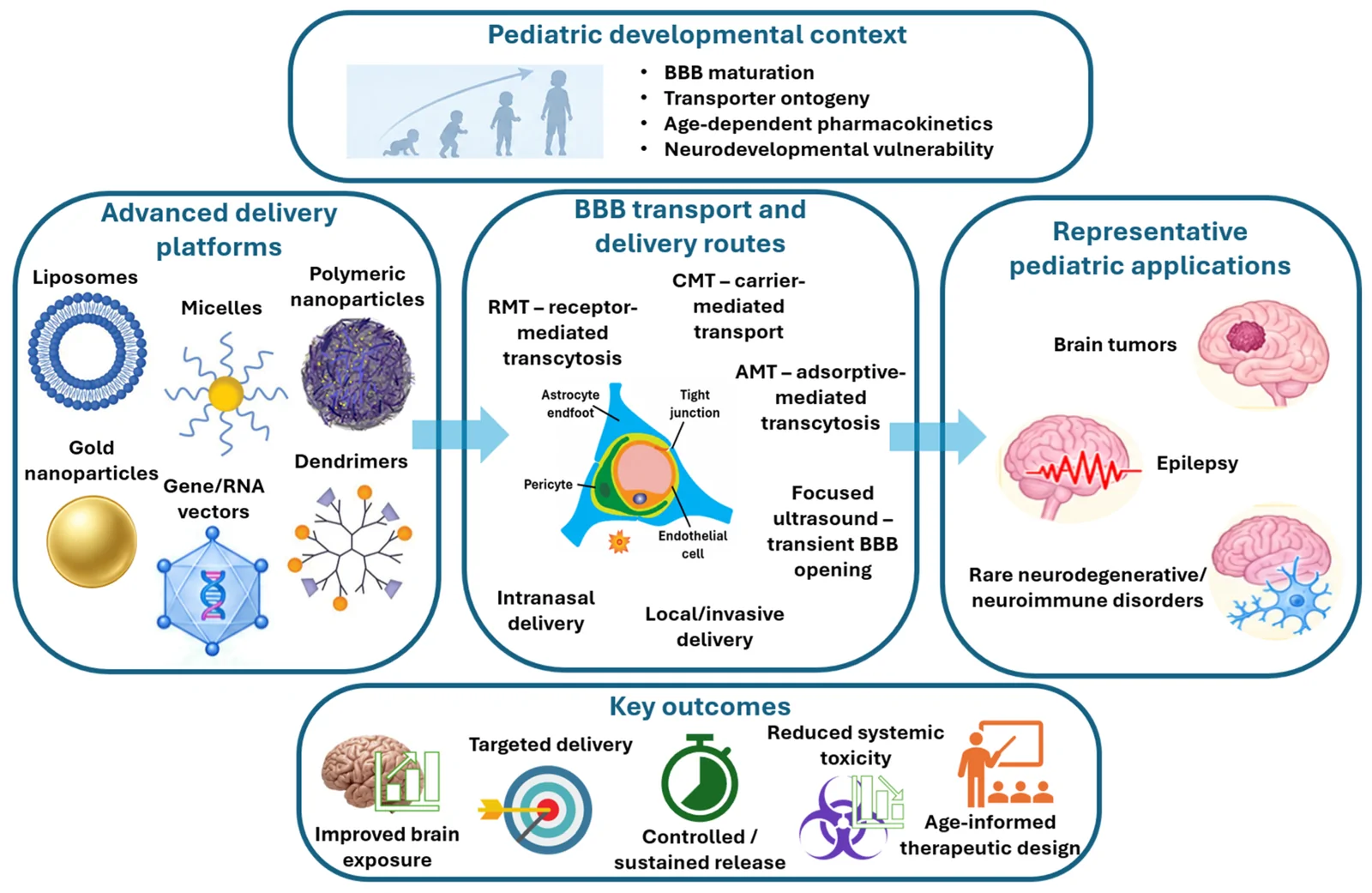
Integrated overview of advanced CNS drug-delivery strategies in pediatric neurology.

Both organic nanoarchitectures, such as liposomes and dendrimers, and inorganic nanomaterials, including gold, silver, and magnetite, have been investigated [97,109,110,111,112]. Their physicochemical properties and surface functionalization with ligands, proteins, or aptamers can modify interactions with the BBB, biodistribution, and targeting specificity [113,114]. Figure 3 provides an integrated overview of the developmental factors, BBB transport mechanisms, advanced delivery platforms, and representative therapeutic applications that shape CNS drug delivery in pediatric patients.

Even though BBB structural and functional properties continue to change during development, there is no clinical evidence to date supporting dimensional thresholds for validated nanoparticles in specific pediatric age groups, such as newborns, infants, or adolescents. Studies performed on human brain tissue demonstrate age- and transporter-dependent maturation, with functional efflux mechanisms demonstrated from fetal development [40,60,61,62]. Thus, the BBB should not be considered a “more permeable” barrier that allows the passage of larger nanoparticles. Particle dimension is one of the factors influencing brain transport, together with surface properties, charge, ligand functionalization, stability in the biological environment, systemic clearance, and transendothelial transport mechanisms. Experimental studies have demonstrated that nanoparticle dimensions influence BBB transport, including when the barrier is opened by focused ultrasound and in in vitro endothelial models [115,116]. Still, these results have not been obtained in pediatric populations and do not allow the definition of age-specific optimal intervals. Consequently, extrapolating dimension thresholds from adults or experimental models to the pediatric population must be undertaken with caution and validated in relevant developmental models.

For a more detailed look at the types of NPs valuable for brain drug delivery, the following subsections discuss liposomes, polymeric NPs, dendrimers, micelles, gold NPs, and gene/RNA therapy tools.

### 3.1. Liposomes

Liposomes are spherical lipid vesicles composed of one or more lipid bilayers, ranging between 50 and 500 nm in diameter, and formed by the emulsification of natural or synthetic fats in an aqueous medium. Their dimensions, dual hydrophobic and hydrophilic nature, biocompatibility, stability, ease of synthesis, high drug-loading efficiency, high bioavailability, and the safe excipients employed in their formulation make liposomes the most widely used NPs in nanomedicine [117,118].

Despite liposomes’ lipophilic characteristics, their size prevents them from easily diffusing across cell membranes or the BBB. Instead, liposomes penetrate the BBB through transport systems, including adsorptive-mediated transcytosis (AMT), receptor-mediated transcytosis (RMT), and carrier-mediated transcytosis (CMT). Consequently, liposome-based approaches can be considered a physiological approach, as the liposome can passively target and accumulate in tumors and at inflammatory sites due to increased permeability and retention. Liposomes can encapsulate chemotherapeutic agents and enable targeted delivery across the BBB. Further addition of polyethylene glycol (PEG) or polysaccharides to the liposome surface creates a protective layer that shields the carrier from plasma protein binding, thereby preventing opsonization and the consequent clearance of liposomes. The most recent and extensively developed liposomal-based strategies that utilize BBB transport systems are cationic liposomes, long-circulating liposomes, and specifically targeted liposomes [119,120,121,122]. Although PEGylation extends circulation time, it does not guarantee effective BBB penetration. This is due to PEGylation’s primary benefits—stealth and decreased immune clearance—rather than its active targeting of BBB transport pathways. Otherwise, liposomes can be modified with active targeting ligands for the specific delivery to brain tumors, including transferrin, folic acid, peptides (such as RGD, Angiopep-2, T7), and monoclonal antibodies. These ligands are attached to the liposome surface to enhance receptor-mediated uptake or facilitate transcytosis across the BBB [123].

### 3.2. Polymeric Nanoparticles

Polymeric NPs are colloidal systems composed of biocompatible, biodegradable, and poorly soluble polymers. These NPs are used to dissolve, encapsulate, adsorb, or chemically bind pharmaceuticals to the polymer matrix and can be fabricated in many forms, sizes, and architectures. Polymeric NPs can be tuned to deliver multiple drugs via hydrophilic, hydrophobic, or electrostatic interactions, as well as via responsive covalent bonds. The simplicity of surface modification using various compounds to attain targeted distribution and prolonged circulation has facilitated the integration of desired features into polymeric NPs. Molecularly imprinted polymer (MIP) NPs are a promising and developing platform for advancing nanomedicine, applicable in delivering medications, cancer therapy, treating neurological diseases, pathogen detection, and identifying biological markers [124,125].

Among the worth-mentioning polymeric NPs that are promising tools for crossing the BBB are the following biodegradable polymers: poly(lactic-co-glycolic acid) (PLGA), poly(caprolactone) (PCL), poly(β-amino ester) (PBAE), chitosan, and albumin [126,127]. Moreover, customized polymeric NPs can retain their contents against enzymatic degradation and ciliary clearance. Drugs administered nasally to the CNS are immediately absorbed through the olfactory and trigeminal pathways, bypassing the BBB, and may have advantageous pharmacokinetics and pharmacodynamics. Although most compounds are unlikely to achieve therapeutic concentrations in the CNS by intranasal administration, polymeric NPs such as chitosan, PLGA, and PLA have been reported to be successful in this delivery approach applied to rats [126,128,129].

### 3.3. Dendrimers

Dendrimers are highly branched, three-dimensional polymeric nanocarriers characterized by nanometric dimensions, well-defined architecture, and a high number of functional groups on their surfaces [130]. Their structure allows both drug encapsulation and surface-level targeting ligand conjugation, while their versatility and possibility of controlled chemical modification permit the adjustment of solubility, stability, drug loading, and interaction with biological barriers [131,132,133].

At the same time, surface properties significantly influence the safety profiles; particularly, cationic charge dendrimers can exhibit cytotoxicity and hemotoxicity, which is why strategies such as PEGylation and other terminal group modifications are used to improve biocompatibility [134,135]. In neurological applications, dendrimers can be functionalized with ligands that favor interaction with the BBB endothelium or with overexpressed receptors in specific brain tumors, facilitating targeted delivery of drugs and genetic material [136,137,138]. Such platforms have been investigated in the context of brain tumors, neurodegenerative disorders, and gene therapy, where they can enhance bioavailability and improve brain distribution of the therapeutic load [136,137,138,139]. Nonetheless, their performance depends on dendrimer generation, surface charge, degree of functionalization, and biodegradability, with the long-term safety and relevance for the pediatric population requiring supplementary evaluation.

### 3.4. Micelles

Polymeric micelles are nanocarriers formed through the self-assembly of amphiphilic copolymers into a core–shell type of structure in which the hydrophobic core can solubilize poorly soluble compounds, while the hydrophilic shell contributes to the system’s stability and circulation prolongation [140,141,142,143,144]. Their reduced dimensions and adjustable surface properties render these systems appealing for CNS-level targeted delivery and for transporting hydrophobic molecules.

Copolymer properties can be tailored to improve BBB permeability and targeting capacity. Pluronics have been particularly investigated for the administration of anti-epileptic drugs, including P-gp modulation. Moreover, micelles carrying multiple drug loadings have demonstrated good tolerability and reduced tumor growth in glioma experimental models, supporting their potential as platforms for combined brain delivery [145,146,147].

### 3.5. Gold Nanoparticles

Gold nanoparticles (GNPs) have attracted interest for targeted drug delivery because of their optical properties, chemical stability, ease of functionalization, and tunable surface characteristics [148,149,150]. The distinctive characteristic of tumor sites facilitates the passive accumulation of GNPs in these areas. Consequently, GNPs can be internalized by cells via non-specific receptor-mediated endocytosis (RME), thereby increasing their presence in the tumor microenvironment [151,152].

GNPs can be loaded or affixed with a variety of therapeutic molecules to enhance the stability and solubility of pharmaceuticals while minimizing undesirable effects. Notably, the association with GNPs can improve the delivery of medicinal drugs into brain tissue [153,154]. Furthermore, due to the robust binding affinity of GNPs for mercaptan, disulfide, and amine, the surfaces of GNPs might be readily modified with diverse functional molecules to enhance their aqueous dispersibility, extend their circulation time in the bloodstream, and impart targeting specificity. These properties support the use of GNPs as versatile carriers for BBB-targeted delivery and for diagnostic and therapeutic applications in brain tumors [155]. A study shows that pulsed laser excitation of vascular-targeted GNPs non-invasively and reversibly modulates the permeability of the blood–brain–tumor barrier (optoBBTB) and improves the delivery of paclitaxel in two genetically engineered female mouse models that replicate two significant glioma phenotypes. This approach significantly reduced tumor volume and extended survival by 50% and 33%, respectively [156,157,158,159].

### 3.6. Gene Therapy Vectors and RNA-Based Delivery Tools

The transfer of DNA/RNA into cells primarily involves two approaches: viral and non-viral vectors. RNA-based technologies, including mRNA substitution therapy, are gaining popularity as intriguing and increasingly feasible therapies. mRNA treatment has significant promise as a mutation-agnostic strategy capable of targeting nearly all monogenic loss-of-function disorders. Therapeutic mRNA encodes a functional version of the faulty protein, thereby circumventing the challenge of targeting specific genetic variants. Furthermore, unlike traditional gene therapy, mRNA-based therapeutics are administered via a simpler process that requires only cytoplasmic delivery, thereby reducing the mutagenesis risks associated with DNA integration. Moreover, mRNA treatment has a temporary effect on target cells, thereby reducing the risk of prolonged, unwanted effects. RNA-based techniques may offer a viable option for drug development in neurodegenerative diseases, as they can target multiple disease circuits [160,161,162].

Viral vectors are potent and widely used for delivering genetic materials, such as DNA and RNA, enabling personalized treatment strategies for conditions like cancer, diabetes, and tuberculosis [162]. Adeno-associated viruses (AAVs) are the most widely used in neurological gene therapy owing to their low immunogenicity, prolonged gene expression, and ability to cross the BBB. AAVs have been effectively utilized in preclinical and clinical studies for conditions such as spinal muscular atrophy (SMA) and PD. Lentiviral vectors, derived from human immunodeficiency virus, offer the advantage of persistent integration into the host genome, making them suitable for prolonged expression in both dividing and non-dividing cells [163,164,165]. Although viral vectors are effective, they are constrained by immunogenicity, limited cargo capacity, and production complexity. Consequently, non-viral transport mechanisms have garnered attention as safer, more secure, and more diverse options for gene therapy in neurological disorders. The non-viral vectors include naked DNA, particle-based, and chemical-based vectors. They are delivered by direct administration (plasmid DNA/naked DNA), chemical methods, or physical techniques [166,167].

## 4. Applications for Advanced Drug Delivery Systems in Pediatric Neurology

The following disease areas illustrate representative applications of advanced CNS drug-delivery technologies across distinct therapeutic and translational contexts in pediatric neurology.

### 4.1. Brain Tumor Treatments with Sustained-Release Nanoplatforms

Given the variability in tumor response to different therapies, accurate classification of CNS tumors is essential for guiding treatment decisions. Supratentorial tumors, located above the tentorium, primarily involve the cerebral hemispheres, whereas infratentorial tumors, situated below the tentorium and hence arising from the brainstem, cerebellum, and fourth ventricle, are distinct. Additionally, the sellar region at the skull base represents another key site for pediatric tumors. Pediatric patients frequently present with high-grade gliomas, low-grade gliomas, embryonal tumors, atypical teratoid/rhabdoid tumors, meningiomas, choroid plexus tumors, and pineal tumors [168,169].

One technique entails the development of prodrugs capable of traversing the BBB, which may be bioconverted into the active drug upon reaching the CNS. These efforts underscore the urgent need for alternative options in pediatric neuro-oncology, with NPs potentially facilitating the development of innovative therapeutic and diagnostic agents. The development of novel NPs for early-stage cancer detection is crucial for extending the therapy window and thereby enhancing prognosis. Currently, the standard radiological imaging method for identifying brain tumors is magnetic resonance imaging (MRI) with gadolinium (Gd) complexes as contrast agents. This method is utilized for surgical planning, navigation, and postoperative evaluations [170]. Effective gadolinium-based scans require a substantial amount of contrast agent; however, concerns about the toxicity of gadolinium complexes have limited certain linear gadolinium agents for MRI body scans, and the European Medicines Agency (EMA) suspended approvals for others in 2017. Likewise, the FDA has restricted the use of gadolinium-containing contrast agents [171,172]. Consequently, nanotechnology-based contrast agents may offer innovative avenues for early and precise diagnosis, while also advancing non-invasive tumor grading. NPs are distinguished by their capacity to target specific sites and to regulate the spatio-temporal release of their payloads. Furthermore, NPs can enhance the reliability, bioavailability, and bioactivity of underutilized drugs, which are affected by their adverse pharmacokinetic properties. In recent years, liposomes, polymeric micelles, dendrimers, and polymeric and inorganic NPs have been effective for brain tumor treatment due to their capacity to traverse the BBB and have demonstrated potential for imaging, drug release, brain cancer targeting, and evasion of the reticuloendothelial system [173,174,175]. For an at-a-glance perspective on theranostic NPs, Table 1 presents some relevant examples for brain cancer management.

Nanoplatforms are actively being explored for pediatric neurology, but most remain in early clinical stages despite promising preclinical results. Ongoing research focuses on long-term safety, age-appropriate dosing, and regulatory approval before wider clinical use [21,108].

### 4.2. Epilepsy Management with Targeted Nanocarriers

Epilepsy represents one of the most frequent neurological conditions, with antiseizure drugs (ASDs) being the primary therapeutic strategy. Regardless, treatment efficacy can be limited by insufficient drug exposure of the brain tissue determined by BBB properties and pharmacoresistance mechanisms, such as increased expression of efflux transporters [185,186,187,188].

This issue is particularly relevant in the pediatric population, where pharmacoresistant epilepsy prevalence is estimated at approximately 25% [189], and the developmental-dependent modifications of pharmacokinetics and BBB transport function can influence brain exposure, efficiency, and the safety profile of the treatment.

Neurocognitive and behavioral adverse effects of antiseizure treatment represent a constant concern in pediatrics, as drug exposure takes place in an active neurodevelopmental stage. The neurocognitive profile differs across drugs and can be influenced by dosage, treatment duration, and patient age, which supports interest in delivery strategies that optimize brain exposure and limit systemic toxicity [190,191].

Pharmacokinetic evidence obtained directly from pediatric patients confirms that ASD exposure is age-dependent and cannot be simply extrapolated from the adult population. Hughes et al. [192] have demonstrated that the optimal levetiracetam regimen for neonatal convulsions must be tailored to target plasma concentrations that may differ from those used in older children and adults.

Li et al. [193] have used pharmacokinetic and pharmacodynamic modeling to recommend maintenance dosing of perampanel for different pediatric conditions. At the same time, Wang et al. [194] developed a population-based pharmacokinetic model to personalize valproic acid dosing in children with epilepsy. These data highlight the importance of age-adapted administration strategies and justify the separate evaluation of advanced drug delivery systems in the pediatric population.

High-frequency neuronal activity characteristic of convulsions can be extrapolated for developing electro-responsive systems for controlled drug release [195,196]. Wu et al. [197] used polyvinyl alcohol as a stabilizer and ammonium persulfate as an oxidant to encapsulate the phenytoin (PHT) sodium drug into polymer NPs, including polypyrrole (PPY) and polydopamine (PDA). The heat generated by photothermal conversion can increase BBB permeability, facilitating the non-invasive delivery of drugs to deeper cerebral regions. Surface functionalization with the brain-targeting peptide angiopep-2 and radiation-induced hyperthermia synergistically enhances drug transport to the brain. During “on–off” cycles of electrical stimulation, the medication is released in the “on” state, but leakage in the “off” state is minimal. This signifies a successful effort to mitigate peripheral adverse responses associated with PHT treatment.

Magnetic-responsive nanodrug delivery systems can facilitate targeted delivery of ASD when an external magnetic field is applied. SPIONs have significant uses in drug administration, MRI, and magnetic hyperthermia [198]. Xu et al. [199] synthesized heparin-modified superparamagnetic iron oxide nanoparticles (UFH-SPIONs) and examined their antiepileptic efficacy and underlying mechanisms. UFH-SPIONs are stable, homogeneous nanosystems that exhibit antioxidant enzyme activity, can traverse the BBB, and accumulate in epileptogenic foci in the hippocampus. Pretreatment with UFH-SPIONs significantly delayed the onset of seizures and diminished their severity following lithium/pilocarpine (LP)-induced seizures in rats. The results indicate that pretreatment with UFH-SPIONs mitigates LP-induced seizures, attenuates the inflammatory response and oxidative stress, and reduces the elevation in apoptotic cells, thereby preserving neuronal function during epilepsy.

Oxidative stress and excessive ROS production contribute to neuronal lesions and the perpetuation of epileptogenesis, offering targets for microenvironment-responsive delivery systems [200,201]. Zhou et al. [202] report the development of a polymeric micelle drug delivery system that facilitates brain targeting and controls the cerebral environment. A phenylboronic ester sensitive to ROS was coupled with PEG to create amphiphilic copolymers. Furthermore, dehydroascorbic acid (DHAA), a glucose analog, was utilized via GLUT1 and enhanced micelle permeation through the BBB. A traditional hydrophobic AED, lamotrigine (LTG), was enclosed within the micelles by self-assembly. Upon administration and translocation across the BBB, ROS-scavenging polymers were anticipated to integrate antioxidative, anti-inflammatory, and neuro-electric regulation strategies into a single approach, offering a promising option for enhancing neuroprotection during the initial stages of epileptogenesis.

Dynamic pH changes in the epileptic focus represent another microenvironment characteristic that can be exploited to develop responsive delivery systems [203,204,205]. Trivedi et al. [206] sought to develop an innovative nanoparticulate theranostic nanocarrier for the dual purposes of diagnosing and treating epilepsy by: i) in situ identification of epileptic conditions via characteristic pH alterations through the synthesis of a pH-responsive polymer (CS-g-PD) and ii) ‘on-demand’ therapeutic prevention of epileptic seizures utilizing a glutaminase inhibitor, 6-diazo-5-oxo-norleucine (DON). In vivo investigations in Wistar rats showed a reduction in epileptic seizures, an increase in latency duration, and a decrease in the period of convulsions and recovery time. Moreover, it effectively decreased glutaminase levels in the brains of PTZ-kindled rats, thereby reducing glutamate levels. Consequently, the nanocarriers have significant promise as ‘on-demand’ theranostics in epilepsy by diminishing both the frequency and intensity of seizures.

For clarity, an at-a-glance summary of the researched nanosystems for epilepsy management is included in Table 2.

Despite the promising experimental results, the pediatric relevance of the above-described nanosystems remains limited by the models in which they have been evaluated. The electro-responsive systems, superparamagnetic nanoparticles, ROS-sensitive micelles, and pH-sensitive nanocarriers reported in studies [197,199,202,206] have been investigated mainly in non-pediatric epilepsy animal models, including pharmacologically induced convulsion models. These studies provide important proof-of-concept evidence for BBB permeability, controlled drug release, reduction in oxidative stress and neuroinflammation, and alleviation of convulsive parameters, but do not directly demonstrate the efficacy and safety of these nanosystems in the developing brain. Thus, their results should be considered preclinical evidence with translational potential, rather than pediatric validation.

Previously discussed pharmacokinetic pediatric data confirm that development can substantially modify the dosage-exposure relation, supporting the need for independent evaluation of nanocarrier performance in the developing brain. Future studies should include neonatal and juvenile models, relevant genetic models of pediatric epileptic encephalopathy, age-dependent evaluations of pharmacokinetics and biodistribution, and long-term monitoring of neurodevelopmental effects.

### 4.3. Nanocarrier Systems for Pediatric Neurodegenerative Disorders

A series of neurodegenerative disorders can also be found in children, despite their recognized predominance in older individuals. Diseases, such as Huntington’s disease (HD), Parkinson’s disease (PD), dementia, and multiple sclerosis (MS), have been increasingly identified in the pediatric population with distinct features compared to adult-onset diseases [207,208,209].

#### 4.3.1. Juvenile Huntington’s Disease

Juvenile Huntington’s disease (JHD), defined by onset before 20 years of age, represents a rare and severe form of HD characterized by more rapid progression, earlier cognitive decline, and distinctive features such as epilepsy, spasticity, ataxia, and liver steatosis, with disease duration inversely correlated to CAG repeat length [210,211].

Unlike adult-onset HD, JHD lacks dedicated pharmacological or multidisciplinary treatment guidelines, and therapeutic strategies remain largely symptomatic. Considering the limited efficiency of conventional antioxidant or anti-protein aggregation-oriented strategies [212,213], several nanosystems have been investigated in HD experimental models for improving bioavailability, passing the BBB, and targeted delivery at the neuronal level. For instance, selenium nanoparticles (SeNPs) have reduced oxidative stress and huntingtin aggregation, while improving neuronal function in transgenic models [212].

Other investigated platforms include solid lipid nanoparticles with rosmarinic acid for intranasal administration [214], citrate-functionalized manganese oxide nanozymes [215], and PEGylated PLGA nanoparticles loaded with epigallocatechin-3-gallate and ascorbic acid [216]. These results support the feasibility of using nanocarriers to modulate relevant mechanisms in HD, but do not demonstrate their efficacy in JHD.

Still, the described studies have been conducted on HD preclinical models that do not specifically reproduce JHD or the developing brain. Consequently, their JHD relevance remains mostly translational and requires validation in relevant juvenile genetic models.

#### 4.3.2. Juvenile Parkinsonism

Juvenile Parkinsonism, defined by symptom onset before 21 years of age, represents a rare and heterogeneous group of disorders distinct from the more prevalent adult-onset PD. While childhood- and adolescent-onset cases are most frequently associated with mutations in genes such as PARK-Parkin, PINK1, and DJ-1, the clinical presentation is often atypical, including dystonia, psychiatric symptoms, cognitive impairment, and additional movement disorders [208,217].

Although levodopa remains the therapeutic mainstay, its long-term use in both adult and pediatric patients is complicated by motor fluctuations and dyskinesias, and no disease-modifying treatments are currently available. In this context, nanosystems have been investigated preclinically as potential strategies to enhance BBB penetration, prolong drug release, and combine dopaminergic delivery with antioxidant or neuroprotective effects [218]. Biodegradable polymeric nanomicelles co-delivering L-DOPA and curcumin demonstrated synergistic antioxidant and neuroprotective properties with high cellular compatibility [219]. At the same time, peptidoglycan monomer-functionalized GNPs exhibited strong binding affinity for L-DOPA and dopamine, enabling efficient drug loading and stability [220]. SeNPs, particularly PVP-functionalized variants, further facilitated dopamine and L-DOPA delivery across the BBB, while exerting intrinsic antioxidant effects at the neuronal site of action [221].

However, the above-described nanosystems have been predominantly evaluated in cellular or preclinical PD models rather than in juvenile genetic or developing brain models. Thus, these results should be interpreted as proof-of-concept evidence of the potential to optimize L-DOPA and dopamine transport and modulate oxidative stress, rather than a demonstration of their efficacy and safety in juvenile Parkinsonism. Validation in relevant juvenile genetic models, with evaluation of pharmacokinetics, biodistribution, and effects on neurodevelopment, is mandatory before extrapolation toward the pediatric population.

#### 4.3.3. Childhood Dementia

Childhood dementia encompasses over 100 rare and ultra-rare monogenic disorders, unified by progressive neurocognitive decline, loss of acquired skills, and severely shortened life expectancy [222,223,224]. Unlike adult dementias, these conditions are under-recognized, partly because of their low prevalence and classification as discrete pathological entities rather than a broader clinical phenotype [222]. With an estimated incidence of 1 in 2800 births and fewer than 5% of cases having disease-modifying therapies, there remains a profound unmet medical need [223].

Nanotechnology has been investigated as a potential strategy for improving brain-specific drug delivery, pharmacokinetics, and targeting while reducing systemic exposure [188,225]. Several nanosystems demonstrate translational potential. For instance, liposomal carriers of *Aphanamixis polystachya* leaf extract showed improved neurobehavioral and anti-inflammatory effects compared to free extract in dementia models [226]; intranasal nanoemulsions of memantine achieved efficient nose-to-brain delivery and sustained antioxidative activity [227]; and nanostructured lipid carriers co-delivering curcumin and donepezil provided synergistic cholinergic, anti-amyloid, and anti-inflammatory effects with superior brain targeting [228]. Likewise, SLNs encapsulating piperine demonstrated superior neuroprotective effects compared with donepezil in experimental AD models [229]. In contrast, dual-functional PEGylated PLA NPs conjugated with BBB-targeting and amyloid plaque-targeting peptides enabled precise drug accumulation at amyloid sites with minimal cytotoxicity [230].

Nonetheless, most of these platforms have been evaluated in experimental models of adult-onset dementia and not in the monogenic diseases accounting for the majority of pediatric dementia. Hence, their relevance for childhood dementia remains mostly translational and necessitates disease- and age-specific validation.

#### 4.3.4. Pediatric-Onset Multiple Sclerosis

MS is among the most severe CNS autoimmune disorders [231], with pediatric-onset MS (POMS; MS beginning before 18 years) showing more active inflammation, frequent relapses, and early cognitive impact than the adult-onset disease, also facing uncertainties regarding the safety and generalizability of disease-modifying therapies in children [207].

Nanotechnology offers strategies to address these gaps by improving CNS delivery, reducing systemic exposure, and targeting complementary pathogenic axes beyond broad immunosuppression [232]. Remyelination-directed approaches include clean-surfaced, faceted gold nanocrystals that promoted oligodendrocyte maturation, upregulated myelin-related genes, enhanced cellular energy metabolism, and restored function in demyelination models, with oral dosing improving motor readouts—supporting a novel remyelinating therapeutic concept for MS [233].

Drug repurposing via nanosystems has likewise shown promise: intranasal teriflunomide nanostructured lipid carriers achieved higher ex vivo permeation and accelerated remyelination with favorable safety in cuprizone mice, demonstrating preclinical feasibility of the nose-to-brain route, although its relevance to pediatric-onset MS remains to be validated in juvenile models and pediatric patients [231]. For systemically administered agents, dimethyl fumarate formulated as tocopherol-acetate nanolipid carriers increased oral bioavailability ~4-fold and substantially improved brain exposure with controlled release and robust cellular uptake, offering a path to a better efficacy–tolerability balance [234]. Immune-targeted nanotherapies further expand the treatment alternatives. For example, curcumin loaded into HDL-mimicking peptide–phospholipid scaffolds (HPPS) selectively engaged scavenger receptor class B type I (SR-B1) on monocytes, curtailed their CNS trafficking, dampened NF-κB signaling, and reduced experimental autoimmune encephalomyelitis (EAE) morbidity from 100% to 30% [235]; antigen-specific tolerance using dual-sized polymeric microparticles abrogated disease in EAE [236], and carrier properties (PLA vs PLG) critically shaped peripheral immune polarization and durable clinical benefit [237]. Finally, pro-remyelinating microRNA delivery—miR-219a-5p via extracellular vesicles outperforming liposomes/PLGA for BBB transit and oligodendrocyte precursor differentiation, improving EAE outcomes, and supporting further investigation of these approaches for POMS rather than demonstrating pediatric efficacy [232,238].

### 4.4. Evidence Gaps and Translational Considerations in Pediatric Nanomedicine

A fundamental limitation of pediatric neurological nanomedicine is the discrepancy between the populations to which these technologies are potentially addressed and the experimental models in which most have been developed. While there are direct pediatric data for pharmacokinetics and clinical utilization of several conventional neurologic drugs [192,193,194] and early pediatric clinical trials for several advanced delivery systems, especially in CNS oncology, a substantial proportion of evidence regarding nanocarriers comes from adult animal models, cell lines, and in vitro systems. These findings are valuable for identifying transport mechanisms and demonstrating technological feasibility, but their translation potential to newborns, children, and adolescents must be presented separately from the evidence obtained directly in the pediatric population.

Extrapolation is particularly limited by the dynamic nature of the BBB and the evolving pharmacology. Primary studies on human brain tissue demonstrated age-dependent modifications of efflux transporter expression and have shown that BBB maturation is not a mere transition from a “permeable” barrier to an “impermeable” one [40,60,61,62]. These differences can alter drug exposure in the brain and influence interactions between nanocarriers and the brain endothelium. Moreover, immune system maturation, modifications in the neurovascular composition and function, and age-dependent systemic pharmacokinetics can impact both the efficacy and safety of a delivery system. Therefore, demonstrating BBB permeability and efficiency in an adult model is not sufficient to infer equivalent pharmacological behavior in the pediatric brain. Importantly, directly comparative pharmacokinetic studies evaluating free drugs and their nanocarrier-formulated counterparts in pediatric patients or age-appropriate juvenile models remain scarce, limiting quantitative assessment of formulation-related changes in systemic and brain exposure [239].

Another important translational problem is long-term safety evaluation. For children-dedicated therapies, exposure can happen in a period of rapid neurodevelopment and can be followed by a life duration of many decades. Consequently, nanoparticle evaluation should go beyond conventional acute toxicity parameters and include tissue persistence and clearance, accumulation following repeated administrations, effects on myelination and the maturation of neuronal circuits, as well as potential late neurobehavioral effects. These aspects are highly important for inorganic or poorly biodegradable nanomaterials.

To fill these gaps, future research should use a stage-wise development strategy which includes: in vitro BBB models relevant for development, neonatal or juvenile animal models adapted to the investigated condition, evaluation of pharmacokinetics and biodistribution according to age, neurotoxicity longitudinal studies, and, later, pediatric clinical studies with adequate age stratification. Such an approach will allow a clearer distinction between platforms that demonstrate only technological feasibility and those that provide sufficient evidence for a realistic pediatric translation.

## 5. Safety and Ethical Considerations

Pediatric formulations must be adapted to age-dependent characteristics, including body weight, swallowing capacity, acceptability, and the need for precise dosing. In addition to efficacy and adequate bioavailability, the safety of excipients and the feasibility of dose-reproducible administration are essential criteria for pediatric use [240,241,242]. These requirements become crucial for advanced delivery systems, in which the properties of the carrier and of auxiliary components can influence both systemic exposure and toxicity profile.

Currently, no clinical studies have assessed the long-term accumulation of inorganic NPs in the pediatric brain. Prolonged retention of NPs in the brain has been clearly demonstrated only in vivo experiments, prompting concern about cumulative neurotoxicity over time. Experimental evidence further indicates that this retention correlates with increasing neurotoxicity and structural brain damage. A recent study demonstrated that repeated exposure to silver NPs resulted in persistent accumulation in the hippocampus of mice, along with lipid peroxidation, ferroptosis, and significant spatial memory deficits that persisted even after a recovery period [243]. Another study suggests that long-term accumulation of iron-containing NPs, particularly magnetite (Fe_3_O_4_), may contribute to neurodegenerative processes that resemble or promote Alzheimer’s disease. Magnetite is a highly reactive iron oxide that can catalyze the formation of ROS via Fenton-like reactions, leading to oxidative stress, protein misfolding, and neuronal injury [244].

Long-term safety represents a very important issue related to administering drugs at the CNS level in the pediatric population, given that exposure can take place in critical developmental periods, yet delayed consequences can become evident for long after finishing treatment. Physiological particularities and child-specific maturation processes can modify the pharmacokinetics, pharmacodynamics, and safety profile of nanoformulations, thereby limiting the direct extrapolation of data obtained from adults to the pediatric population [107,239].

Thus, safety evaluation should go beyond analyzing acute cytotoxicity and short-term tolerability and should include biodistribution after repeated administration, tissue accumulation, degradation and elimination of the nano-carrier and its degradation products, and the possibility of material persistence in the CNS or peripheral organs [239,245]. Nanocarrier physicochemical properties, including size, composition, and surface characteristics, influence pharmacokinetics, biodistribution, and clearance. Therefore, the long-term safety profile must be evaluated on a platform-specific basis and cannot be generalized across all nanomaterials [245].

Developmental neurotoxicity represents another important dimension of safety evaluation. The developing brain can exhibit a different vulnerability to nanomaterial exposure than the adult brain, and experimental data on engineered nanomaterials indicate the need for specific evaluation of developmental neurotoxicity, including effects on neuronal function, oxidative stress, inflammation, and neurobehavioral outcomes [246]. Consequently, studies using juvenile models should include, in addition to conventional toxicity parameters, longitudinal assessments of neurobehavioral and cognitive functions, neuropathology, and neuroinflammation, as well as parameters relevant to nervous system maturation, which should be correlated with age-dependent pharmacokinetics and biodistribution [239,246]. In repeatedly administered therapies, it should be established whether exposure leads to progressive accumulation, whether an equilibrium is reached between distribution and elimination, and whether potential adverse effects persist or appear after treatment interruption [245].

These aspects have implications for long-term clinical monitoring. Considering physiological and developmental characteristics in the pediatric population and the limited data on the pharmacokinetics and safety of nanoformulations in children, clinical evaluation must be adapted to age and platform characteristics [107,239]. For systems intended for repeated or chronic administration, longitudinal follow-up should therefore consider delayed neurological, neurocognitive, developmental, and immune-related effects, particularly when the carrier is expected to exhibit prolonged tissue persistence.

Moreover, performing clinical trials in neonates and children is challenging due to the typically limited availability of patients with the condition of interest, the need to enroll across the pediatric age spectrum, and recruitment difficulties. Early-phase studies aimed at characterizing the pharmacokinetic and pharmacodynamic features of a medication in the pediatric population can be challenging due to the small blood volume in newborns, necessitating meticulous planning of the quantity and timing of blood samples [247].

International regulations, including the ICH E11 framework and requirements adopted by agencies such as the EMA and the FDA, aim to integrate pediatric populations into drug development through age-adapted evaluations of pharmacokinetics, efficacy, and safety [248,249,250]. For advanced delivery systems, these requirements are highly relevant because pediatric evaluation must consider both the drug delivered and the carrier’s characteristics, biodistribution, and potential long-term effects.

Table 3 summarizes interventional clinical studies involving nanoparticle- or nanocarrier-based approaches in pediatric brain tumors registered in ClinicalTrials.gov (data accessed 13 August 2026).

In clinical research, participants undergo specific efforts, risks, and limitations in treatment individualization to facilitate the acquisition of new knowledge. A favorable correlation of anticipated benefits and risks, along with free, informed agreement, properly justifies this in adults. Children are not permitted to provide autonomous consent; they may only offer assent, which, together with their legal guardians’ approval, is an unequivocal condition for participation in the study. In some, but not all, nations, oral and written approval from both legal guardians is required [251]. The EMA has recognized recruitment as a particular difficulty in pediatric clinical studies. The present approach involves recruiting at referral sites where patients typically must travel to the institution. Recruitment rates exhibit significant variability, yet a primary justification for the formation of clinical research networks is to facilitate the timely completion of studies. A significant recruiting challenge is the insufficient facilities for pediatric clinical trials, such as the absence of research personnel trained in child-friendly practices within a family-centered environment to enhance recruitment efforts [252].

Pediatric drug development remains challenging, particularly in neurology, where age-dependent pharmacokinetics, limited clinical datasets, and safety considerations complicate dose selection and regulatory approval. Quantitative clinical pharmacology, including pharmacokinetic/pharmacodynamic (PK/PD) modeling, may support age-appropriate exposure–response characterization and facilitate the clinical translation of advanced drug-delivery systems [9,253].

## 6. Future Directions and Conclusions

In pediatric neurology, future delivery strategies should integrate molecular diagnosis with age-dependent pharmacological characteristics. Genetic information and biomarkers can help identify therapeutic targets, yet the systems’ design must also account for BBB maturation, pharmacokinetics, biodistribution, and neurodevelopmental vulnerability [254,255]. This approach could facilitate the generation of platforms tailored to both disease mechanisms and patient developmental stages.

Artificial intelligence and machine learning methods can contribute to future development of drug delivery systems by integrating formulation properties with pharmacokinetic, pharmacodynamic, and preclinical data. In the pediatric context, such tools can be used to optimize carrier characteristics and predict exposure, biodistribution, and toxicity by age; however, their applicability depends on the availability of adequate pediatric data and requires experimental validation [256].

Emerging preclinical nanoneuromodulation platforms may combine remotely controlled neuronal modulation with neuroprotective functions. Ren et al. [257] developed a TiS_2_/Pt nanotransducer capable of wireless modulation of neuronal electrical activity while reducing oxidative stress and seizure severity in experimental epilepsy models; however, its safety, developmental effects, and clinical applicability in pediatric epilepsy remain unknown [258].

Advanced delivery systems offer the potential to improve brain exposure, reduce systemic toxicity, and facilitate targeted delivery of drugs, nucleic acids, and other therapeutics in pediatric neurological conditions. Nonetheless, for most of the nanocarriers analyzed in this review, available evidence remains predominantly derived from non-pediatric preclinical models, and their demonstrated efficacy in adults or in experimental models cannot be directly extrapolated to the developing brain. Clinical translation will require relevant neonatal and juvenile models, characterization of pharmacokinetics and biodistribution by age, toxicity evaluation following repeated administration, and monitoring long-term neurodevelopmental effects. Prioritizing these objectives will be essential for differentiating platforms with technological feasibility from those that can demonstrate a safe clinical, reproducible benefit in the pediatric population.

## Figures and Tables

**Table 1:** Research on theranostic NPs examined for brain cancer that integrates a therapeutic anti-cancer action with tumor imaging.

NP Category		Functional Components	Model	Results	Refs.
Liposomes	Engineered HDL-mimetic nanoparticles (eHNPs)	Apolipoprotein A1, anti-CD15, and SHH subtype of MB inhibitor—LDE225	Mice (SHH MB cells)	Traverse the BBB in SHH MB in vivo and administer the payload efficiently to all disease locations in a highly tailored manner.	[176]
Polymeric NPs	PBAE NPs	HSVtk	Mice (D425 MB and BT-12 atypical teratoid/rhabdoid tumors—AT/RT cell line)	NPs enabled 45% transfection in BT-12 cells but caused>65% cell death when used to deliver HSVtk.	[177]
	PLGA tailored with anti-SSTR2 peptide	3,3′-diindolylmethane	Adult SD rats	Nanoformulation offers a potential therapeutic strategy for glioma, capable of efficiently crossing the BBB and exerting apoptotic and antitumorigenic effects.	[178]
Magnetic NPs	Magnetic IONPs stabilized with TSEDTA	DOX	Mice (GBM U251 cells)	Effective in apoptotic-induced GBM cell death (over 90%) within 48 h of treatment and tumor suppression.	[179]
	IONPs	Paclitaxel	Mice (U251 cells and HMC3 cells)	Suppressed the tumor volume of GBM xenografts and decreased the expression level of GPX4 protein in tumor tissues in comparison with the control group.	[180]
GNPs	PEGylated GNPs covalently conjugated with CD133 aptamer (GNPs-PEG-CD133-CB-839)	GLS1 inhibitor Telaglenastat (CB-839)	In vitro (CD133-positive brain tumor models)	Significantly decreased the viability of CD133-positive cancer cells in a dose-dependent manner.	[181]
	Ultrasmall GNPs	DOX and AlexaFluor647	Mice (U87 cells)	Inhibited brain tumor growth and prolonged animal survival without adverse side effects. Most of the NPs (84%) had been excreted from the animal after 24 h.	[182]
Dendrimers	Poly(amidoamine) dendrimer-based carrier functionalized with Ang2 and EGFR-targeting peptide (EP-1)	DOX	Mice (U87-MG cells)	Exhibited outstanding BBB penetrability and enhanced the anti-glioma effect of the drugs and prolonged the survival of glioma-bearing mice.	[183]
	Biotinylated G3 dendrimers	Celecoxib	In vitro (U-118 MG glioma cells) and Roundworm (*Caenorhabditis elegans*)	Improved apoptosis of GBM cells in vitro and reduced proliferation compared to administration of the free drug alone.	[184]

Abbreviations: APP—amyloid precursor protein; TMZ—Temozolomide; GBM—Glioblastoma; SHH—sonic hedgehog; MB—medulloblastoma; PBAE—Poly(beta-amino ester); HSVtk—Herpes Simplex Virus type I thymidine kinase; IONPs—iron oxide nanoparticles; TSEDTA—trimethoxysilylpropyl-ethylenediamine triacetic acid; DOX—doxorubicin; Ang2—angiopep-2; EGFR—epidermal growth factor receptor.

**Table 2:** Research on nanosystems examined for epilepsy management.

NP Category		Functional Components	Model	Administration Route	Target Receptor/Targeting Mechanism	Results	Refs.
Polymeric NPs	Nanomicelles composed of PPY and PDA	Phenytoin sodium drug	Male mice and male SD rats	Intraperitoneal	LRP1 via Angiopep-2; NIR-assisted BBB penetration	Enabled controlled on–off PHT release at 50 μA, improved BBB penetration via photothermal and Angiopep-2 effects, and achieved sustained delivery with minimal off-state leakage	[197]
	Micelles made of ROS-sensitive phenylboronic ester and PEG, functionalized with DHAA	LTG	Male SD rats	Intravenous (tail vein)	GLUT1 via DHAA	Crossed the BBB via GLUT1 targeting, enabling brain-specific delivery. The system combined antioxidant, anti-inflammatory, and neuroprotective actions, enhancing LTG distribution and antiepileptic efficacy.	[202]
	Theranostic nanocarrier made of a pH-responsive polymer (CS-g-PD)	DON	Wistar rats	Intranasal	No receptor-mediated targeting; pH-responsive, nose-to-brain delivery	Enabled drug release at acidic pH (6.4) and inhibition at pH 7.2. In vivo, they reduced seizure frequency and duration, increased latency, and lowered brain glutaminase and glutamate levels, demonstrating effective on-demand theranostic activity.	[206]
Magnetic NPs	Heparin-modified SPIONs	Heparin	Rats with LP-induced seizures	Intravenous (tail vein)	No specific receptor-mediated targeting reported	UFH-SPIONs crossed the BBB and accumulated in hippocampal foci, delaying seizure onset and reducing severity in LP-induced epilepsy. Pretreatment decreased IL-6, IL-1β, TNF-α, MDA, and neuronal apoptosis, while increasing SOD and CAT levels, indicating antioxidant, anti-inflammatory, and neuroprotective effects.	[199]

Abbreviations: PPY—polypyrrole; PDA—polydopamine; SPIONs—superparamagnetic iron oxide nanoparticles; LP—lithium/pilocarpine; LTG—lamotrigine; GLUT1—glucose transporter 1; DHAA—dehydroascorbic acid; DON—6-diazo-5-oxo-norleucine.

**Table 3:** Interventional clinical studies using NPs in pediatric brain tumors.

ClinicalTrials.gov Identifier	Type of Cancer	Intervention/ Treatment	Administration Route	Recruitment Status	Phase	Ages Eligible for Study	Last Update Posted	Study Completion (Estimated/Actual)
NCT00019630	BT	Doxorubicin-loaded liposomes	Intravenous	Completed	1	Up to 21 years	4 March 2024	n/r
NCT02013336	BN	Irinotecan-loaded liposomes	Intravenous	Recruiting	1	1 year to 20 years	31 March 2022	December 2023
NCT00003073	CNST	Cytarabine-loaded liposomes	Intrathecal	Unknown status	1	1 year to 21 years	23 March 2010	n/r
NCT03566199	DIPG	Panobinostat Nanoparticle Formulation MTX110	Intratumoral	Completed	1/2	2 years to 21 years	25 February 2025	31 March 2021
NCT04264143	DIPG, DP, TG, DMG	Infusate with MTX110 and gadolinium	Intratumoral	Completed	1	3 years to 18 years	18 December 2023	22 November 2023
NCT00978562	BN	DSC-MRI with ferumoxytol (small iron particles)	Intravenous	Completed	Early 1	5 years to 18 years	3 August 2022	December 2018
NCT04573140	PHGG	RNA-Lipid Particle (RNA-LP) Vaccines	Intravenous	Recruiting	1	4 years and older	25 June 2026	1 July 2029

Abbreviations: n/r—not reported; BT—brain tumor; BN—brain neoplasm; CNST—central nervous system tumors; DIPG—diffuse intrinsic pontine glioma; DP—diffuse pontine; TG—thalamic gliomas; DMG—diffuse midline glioma; PHGG—pediatric high-grade gliomas. Recruitment status and completion dates are reported as listed in ClinicalTrials.gov at the date of access; some older records have not been updated recently and may therefore not reflect the current operational status of the study.

## Data Availability

No new data were created or analyzed in this study. Data sharing is not applicable to this article.

## References

[1] Kanmounye U.S., Abu-Bonsrah N., Shlobin N., Djoutsop O. (2022). Letter: The World Health Organization’s Intersectoral Global Action Plan on Epilepsy and Other Neurological Disorders 2022–2031. Neurosurgery.

[2] Zhang C., Yang X., Wan D., Ma Q., Yin P., Zhou M., Hao J. (2025). Burden of neurological disorders in China and its provinces, 1990–2021: Findings from the global burden of disease study 2021. Med.

[3] Newton C.R. (2018). Global Burden of Pediatric Neurological Disorders. Semin. Pediatr. Neurol..

[4] Domingues C., Jarak I., Veiga F., Dourado M., Figueiras A. (2023). Pediatric Drug Development: Reviewing Challenges and Opportunities by Tracking Innovative Therapies. Pharmaceutics.

[5] Chandramouli M., Vrushabendra B., Srikantamurthy N. (2024). A Comprehensive Review of Pediatric Drug Development: An Extensive Analysis of Present Difficulties and Prospects for the Future. Biointerface Res. Appl. Chem..

[6] Khan T.B. (2025). Advancements and challenges in pediatric pharmacotherapy: A comprehensive review. Glob. J. Health Sci. Res..

[7] Rose K., Grant-Kels J.M. (2019). The Meanings of “Pediatric Drug Development”. Ther. Innov. Regul. Sci..

[8] Espinoza J.C. (2021). The Scarcity of Approved Pediatric High-Risk Medical Devices. JAMA Netw. Open.

[9] Cruz E.D., Rahim F., Lemmon M., Mikati M.A. (2022). US Food and Drug Administration Facilitated Pediatric Approval Programs: Application to Pediatric Neurological Disorders. J. Child Neurol..

[10] Haigo H., Matsuda K., Shikano M. (2025). Clinical data required for the approval of pediatric pharmaceuticals in Japan. BMC Pediatr..

[11] Coelho-Santos V., Shih A.Y. (2020). Postnatal development of cerebrovascular structure and the neurogliovascular unit. Wiley Interdiscip. Rev. Dev. Biol..

[12] Slaoui L., Gilbert A., Rancillac A., Delaunay-Piednoir B., Chagnot A., Gerard Q., Letort G., Mailly P., Robil N., Gelot A. (2023). In mice and humans, brain microvascular contractility matures postnatally. Brain Struct. Funct..

[13] Klostranec J.M., Krings T. (2022). Cerebral neurovascular embryology, anatomic variations, and congenital brain arteriovenous lesions. J. NeuroInterventional Surg..

[14] Abbott N.J., Patabendige A.A.K., Dolman D.E.M., Yusof S.R., Begley D.J. (2010). Structure and function of the blood–brain barrier. Neurobiol. Dis..

[15] Daneman R., Prat A. (2015). The blood-brain barrier. Cold Spring Harb. Perspect. Biol..

[16] Tumani H., Huss A., Bachhuber F., Deisenhammer F., Teunissen C.E., Tumani H. (2018). Chapter 2—The cerebrospinal fluid and barriers—anatomic and physiologic considerations. Handbook of Clinical Neurology.

[17] Obermeier B., Daneman R., Ransohoff R.M. (2013). Development, maintenance and disruption of the blood-brain barrier. Nat. Med..

[18] Power E.A., Rechberger J.S., Gupta S., Schwartz J.D., Daniels D.J., Khatua S. (2022). Drug delivery across the blood-brain barrier for the treatment of pediatric brain tumors—An update. Adv. Drug Deliv. Rev..

[19] Schmitt G., Parrott N., Prinssen E., Barrow P. (2017). The great barrier belief: The blood–brain barrier and considerations for juvenile toxicity studies. Reprod. Toxicol..

[20] Gao Q., Song D., Xu D., Chai X., Ge M. (2025). Characteristics of the blood–brain barrier in pediatric brain tumors. Front. Pediatr..

[21] Hari P., Vahab S.A., Kumar V.S. (2025). Advancements in Liposomal Drug Delivery Systems for Paediatric Neurological Disorders: A Comprehensive Review. AAPS PharmSciTech.

[22] Phoenix T.N., Patmore D.M., Boop S., Boulos N., Jacus M.O., Patel Y.T., Roussel M.F., Finkelstein D., Goumnerova L., Perreault S. (2016). Medulloblastoma Genotype Dictates Blood Brain Barrier Phenotype. Cancer Cell.

[23] Archer T.C., Mahoney E.L., Pomeroy S.L. (2017). Medulloblastoma: Molecular Classification-Based Personal Therapeutics. Neurotherapeutics.

[24] Arvanitis C.D., Ferraro G.B., Jain R.K. (2020). The blood-brain barrier and blood-tumour barrier in brain tumours and metastases. Nat. Rev. Cancer.

[25] Fang J., Nakamura H., Maeda H. (2011). The EPR effect: Unique features of tumor blood vessels for drug delivery, factors involved, and limitations and augmentation of the effect. Adv. Drug Deliv. Rev..

[26] Lu C.T., Zhao Y.Z., Wong H.L., Cai J., Peng L., Tian X.Q. (2014). Current approaches to enhance CNS delivery of drugs across the brain barriers. Int. J. Nanomed..

[27] Haumann R., Videira J.C., Kaspers G.J.L., van Vuurden D.G., Hulleman E. (2020). Overview of Current Drug Delivery Methods Across the Blood–Brain Barrier for the Treatment of Primary Brain Tumors. CNS Drugs.

[28] Pardridge W.M. (2015). Targeted delivery of protein and gene medicines through the blood-brain barrier. Clin. Pharmacol. Ther..

[29] Mueller T., Stucklin A.S.G., Postlmayr A., Metzger S., Gerber N., Kline C., Grotzer M., Nazarian J., Mueller S. (2020). Advances in Targeted Therapies for Pediatric Brain Tumors. Curr. Treat. Options Neurol..

[30] Shellhaas R.A., deVeber G., Bonkowsky J.L., Augustine E.F., Bassuk A.G., Calame D.G., Carrasco M., Dlamini N., Felling R.J., Glass H.C. (2021). Gene-Targeted Therapies in Pediatric Neurology: Challenges and Opportunities in Diagnosis and Delivery. Pediatr. Neurol..

[31] Hall C.N., Reynell C., Gesslein B., Hamilton N.B., Mishra A., Sutherland B.A., O’Farrell F.M., Buchan A.M., Lauritzen M., Attwell D. (2014). Capillary pericytes regulate cerebral blood flow in health and disease. Nature.

[32] Alarcon-Martinez L., Villafranca-Baughman D., Quintero H., Kacerovsky J.B., Dotigny F., Murai K.K., Prat A., Drapeau P., Di Polo A. (2020). Interpericyte tunnelling nanotubes regulate neurovascular coupling. Nature.

[33] Cornelissen F.M.G., Markert G., Deutsch G., Antonara M., Faaij N., Bartelink I., Noske D., Vandertop W.P., Bender A., Westerman B.A. (2023). Explaining Blood–Brain Barrier Permeability of Small Molecules by Integrated Analysis of Different Transport Mechanisms. J. Med. Chem..

[34] Stiles J., Jernigan T.L. (2010). The Basics of Brain Development. Neuropsychol. Rev..

[35] Budday S., Steinmann P., Kuhl E. (2015). Physical biology of human brain development. Front. Cell. Neurosci..

[36] Weller R.O., Sharp M.M., Christodoulides M., Carare R.O., Møllgård K. (2018). The meninges as barriers and facilitators for the movement of fluid, cells and pathogens related to the rodent and human CNS. Acta Neuropathol..

[37] Ballabh P., Hu F., Kumarasiri M., Braun A., Nedergaard M. (2005). Development of Tight Junction Molecules in Blood Vessels of Germinal Matrix, Cerebral Cortex, and White Matter. Pediatr. Res..

[38] Goasdoué K., Miller S.M., Colditz P.B., Björkman S.T. (2017). Review: The blood-brain barrier; protecting the developing fetal brain. Placenta.

[39] Ballabh P., Braun A., Nedergaard M. (2004). The blood-brain barrier: An overview: Structure, regulation, and clinical implications. Neurobiol. Dis..

[40] Lam J., Baello S., Iqbal M., Kelly L.E., Shannon P.T., Chitayat D., Matthews S.G., Koren G. (2015). The ontogeny of P-glycoprotein in the developing human blood-brain barrier: Implication for opioid toxicity in neonates. Pediatr. Res..

[41] Vorbrodt A.W., Dobrogowska D.H., Tarnawski M. (2001). Immunogold study of interendothelial junction-associated and glucose transporter proteins during postnatal maturation of the mouse blood-brain barrier. J. Neurocytol..

[42] Engelhardt B., Liebner S. (2014). Novel insights into the development and maintenance of the blood–brain barrier. Cell Tissue Res..

[43] Saunders N.R., Dziegielewska K.M., Møllgård K., Habgood M.D. (2018). Physiology and molecular biology of barrier mechanisms in the fetal and neonatal brain. J. Physiol..

[44] Zlokovic B.V. (2011). Neurovascular pathways to neurodegeneration in Alzheimer’s disease and other disorders. Nat. Rev. Neurosci..

[45] Andreone B.J., Chow B.W., Tata A., Lacoste B., Ben-Zvi A., Bullock K., Deik A.A., Ginty D.D., Clish C.B., Gu C. (2017). Blood-Brain Barrier Permeability Is Regulated by Lipid Transport-Dependent Suppression of Caveolae-Mediated Transcytosis. Neuron.

[46] Matsumoto J., Stewart T., Sheng L., Li N., Bullock K., Song N., Shi M., Banks W.A., Zhang J. (2017). Transmission of α-synuclein-containing erythrocyte-derived extracellular vesicles across the blood-brain barrier via adsorptive mediated transcytosis: Another mechanism for initiation and progression of Parkinson’s disease?. Acta Neuropathol. Commun..

[47] Gaengel K., Genové G., Armulik A., Betsholtz C. (2009). Endothelial-Mural Cell Signaling in Vascular Development and Angiogenesis. Arterioscler. Thromb. Vasc. Biol..

[48] Armulik A., Genové G., Mäe M., Nisancioglu M.H., Wallgard E., Niaudet C., He L., Norlin J., Lindblom P., Strittmatter K. (2010). Pericytes regulate the blood–brain barrier. Nature.

[49] Sugiyama S., Sasaki T., Tanaka H., Yan H., Ikegami T., Kanki H., Nishiyama K., Beck G., Gon Y., Okazaki S. (2023). The tight junction protein occludin modulates blood–brain barrier integrity and neurological function after ischemic stroke in mice. Sci. Rep..

[50] Keaney J., Campbell M. (2015). The dynamic blood–brain barrier. FEBS J..

[51] Hashimoto Y., Campbell M., Tachibana K., Okada Y., Kondoh M. (2021). Claudin-5: A Pharmacological Target to Modify the Permeability of the Blood–Brain Barrier. Biol. Pharm. Bull..

[52] Yuan S., Liu K.J., Qi Z. (2020). Occludin regulation of blood-brain barrier and potential therapeutic target in ischemic stroke. Brain Circ..

[53] Li Y., Wei J.-Y., Liu H., Wang K.-J., Jin S.-N., Su Z.-K., Wang H.-J., Shi J.-X., Li B., Shang D.-S. (2022). An oxygen-adaptive interaction between SNHG12 and occludin maintains blood-brain barrier integrity. Cell Rep..

[54] Kadry H., Noorani B., Cucullo L. (2020). A blood–brain barrier overview on structure, function, impairment, and biomarkers of integrity. Fluids Barriers CNS.

[55] Guo L., Zhang H., Hou Y., Wei T., Liu J. (2016). Plasmalemma vesicle-associated protein: A crucial component of vascular homeostasis. Exp. Ther. Med..

[56] Shue E.H., Carson-Walter E.B., Liu Y., Winans B.N., Ali Z.S., Chen J., Walter K.A. (2008). Plasmalemmal Vesicle Associated Protein-1 (PV-1) is a marker of blood-brain barrier disruption in rodent models. BMC Neurosci..

[57] Sivaraj K.K., Li R., Albarran-Juarez J., Wang S., Tischner D., Grimm M., Swiercz J.M., Offermanns S., Wettschureck N. (2015). Endothelial Gα_q/11_ is required for VEGF-induced vascular permeability and angiogenesis. Cardiovasc. Res..

[58] Miao Z., Dong Y., Fang W., Shang D., Liu D., Zhang K., Li B., Chen Y.-H. (2014). VEGF Increases Paracellular Permeability in Brain Endothelial Cells via Upregulation of EphA2. Anat. Rec..

[59] Fajardo-Fregoso B.F., Castañeda-Cabral J.L., Beas-Zárate C., Ureña-Guerrero M.E. (2020). Neonatal excitotoxicity modifies blood-brain barrier properties increasing its susceptibility to hypertonic shock in adulthood. Int. J. Dev. Neurosci..

[60] Virgintino D., Errede M., Girolamo F., Capobianco C., Robertson D., Vimercati A., Serio G., Di Benedetto A., Yonekawa Y., Frei K. (2008). Fetal blood-brain barrier P-glycoprotein contributes to brain protection during human development. J. Neuropathol. Exp. Neurol..

[61] Verscheijden L.F.M., van Hattem A.C., Pertijs J., de Jongh C.A., Verdijk R.M., Smeets B., Koenderink J.B., Russel F.G.M., de Wildt S.N. (2020). Developmental patterns in human blood-brain barrier and blood-cerebrospinal fluid barrier ABC drug transporter expression. Histochem. Cell Biol..

[62] Lye P., Bloise E., Imperio G.E., Chitayat D., Matthews S.G. (2022). Functional Expression of Multidrug-Resistance (MDR) Transporters in Developing Human Fetal Brain Endothelial Cells. Cells.

[63] Agarwal S., Hartz A.M.S., Elmquist W.F., Bauer B. (2011). Breast Cancer Resistance Protein and P-Glycoprotein in Brain Cancer: Two Gatekeepers Team Up. Curr. Pharm. Des..

[64] Schäfer A.M., Meyer Zu Schwabedissen H.E., Grube M. (2021). Expression and Function of Organic Anion Transporting Polypeptides in the Human Brain: Physiological and Pharmacological Implications. Pharmaceutics.

[65] Ghosh C., Puvenna V., Gonzalez-Martinez J., Janigro D., Marchi N. (2011). Blood-Brain Barrier P450 Enzymes and Multidrug Transporters in Drug Resistance: A Synergistic Role in Neurological Diseases. Curr. Drug Metab..

[66] Ghosh C., Gonzalez-Martinez J., Hossain M., Cucullo L., Fazio V., Janigro D., Marchi N. (2010). Pattern of P450 expression at the human blood-brain barrier: Roles of epileptic condition and laminar flow. Epilepsia.

[67] Jovanović M., Vučićević K. (2022). Pediatric pharmacokinetic considerations and implications for drug dosing. Arh. ZA Farm..

[68] Mahmood I. (2014). Dosing in Children: A Critical Review of the Pharmacokinetic Allometric Scaling and Modelling Approaches in Paediatric Drug Development and Clinical Settings. Clin. Pharmacokinet..

[69] Job K.M., Gamalo M., Ward R.M. (2019). Pediatric Age Groups and Approach to Studies. Ther. Innov. Regul. Sci..

[70] Batchelor H.K., Marriott J.F. (2015). Paediatric pharmacokinetics: Key considerations. Br. J. Clin. Pharmacol..

[71] Van Den Anker J., Reed M.D., Allegaert K., Kearns G.L. (2018). Developmental Changes in Pharmacokinetics and Pharmacodynamics. J. Clin. Pharmacol..

[72] Kearns G.L., Abdel-Rahman S.M., Alander S.W., Blowey D.L., Leeder J.S., Kauffman R.E. (2003). Developmental pharmacology--drug disposition, action, and therapy in infants and children. N. Engl. J. Med..

[73] Bartelink I.H., Rademaker C.M.A., Schobben A.F.A.M., van den Anker J.N. (2006). Guidelines on Paediatric Dosing on the Basis of Developmental Physiology and Pharmacokinetic Considerations. Clin. Pharmacokinet..

[74] Heinen F., Desloovere K., Schroeder A.S., Berweck S., Borggraefe I., Van Campenhout A., Andersen G.L., Aydin R., Becher J.G., Bernert G. (2010). The updated European Consensus 2009 on the use of Botulinum toxin for children with cerebral palsy. Eur. J. Paediatr. Neurol..

[75] Haché M., Swoboda K.J., Sethna N., Farrow-Gillespie A., Khandji A., Xia S., Bishop K.M. (2016). Intrathecal Injections in Children with Spinal Muscular Atrophy. J. Child. Neurol..

[76] Schreiner T.G., Menéndez-González M., Schreiner O.D., Ciobanu R.C. (2025). Intrathecal Therapies for Neurodegenerative Diseases: A Review of Current Approaches and the Urgent Need for Advanced Delivery Systems. Biomedicines.

[77] Mateos M.K., Marshall G.M., Barbaro P.M., Quinn M.C.J., George C., Mayoh C., Sutton R., Revesz T., Giles J.E., Barbaric D. (2021). Methotrexate-related central neurotoxicity: Clinical characteristics, risk factors and genome-wide association study in children treated for acute lymphoblastic leukemia. Haematologica.

[78] Zhang Y.T., Wang Y., Zhong X.D., Chang J. (2023). Efficacy of intrathecal methotrexate in children with high-risk medulloblastoma over three years: A retrospective study from a single center. J. Neuro-Oncol..

[79] Held A., De Laurentis C., Mietton C., Vuillerot C., Dohin J., Lebedel N., Valayer E.C., Gordon X., Joubaud F., Di Rocco F. (2026). Intrathecal Baclofen Therapy in Children: Testing, Dose-Finding, and Complications—A Single-Center Experience. Pediatr. Neurol..

[80] Hasnat M.J., Rice J.E. (2015). Intrathecal baclofen for treating spasticity in children with cerebral palsy. Cochrane Database Syst. Rev..

[81] Bektaş Ö., Gülşen M., Dursun O.B., Tekin A., Yüksel D., Demir E., Öztürk G., Saltık S., Hergüler Ö., Özçelik A.A. (2025). Nusinersen for children with type I spinal muscular atrophy: 4 years’ clinical experience in Turkish cohort. Front. Neurol..

[82] Li W., Zhang Q., Miao H., Xu J. (2025). Real-world analysis of the efficacy and safety of nusinersen in pediatric patients with spinal muscular atrophy. Orphanet J. Rare Dis..

[83] Stolte B., Totzeck A., Kizina K., Bolz S., Pietruck L., Mönninghoff C., Guberina N., Oldenburg D., Forsting M., Kleinschnitz C. (2018). Feasibility and safety of intrathecal treatment with nusinersen in adult patients with spinal muscular atrophy. Ther. Adv. Neurol. Disord..

[84] Lin W.-S. (2026). Rethinking Corticosteroid Therapy in Pediatric Neurology. J. Inflamm. Res..

[85] Shock M., Nigro E., Donner E.J., Whitney R. (2026). CLN2 Disease: Current Understandings, Challenges, and Future Directions. J. Child. Neurol..

[86] Kim A., Grover A., Hammon K., De Hart G., Slasor P., Cherukuri A., Ajayi T., Jacoby D., Schulz A., Specchio N. (2021). Clinical Pharmacokinetics and Pharmacodynamics of Cerliponase Alfa, Enzyme Replacement Therapy for CLN2 Disease by Intracerebroventricular Administration. Clin. Transl. Sci..

[87] Bharucha-Goebel D.X., Todd J.J., Saade D., Norato G., Jain M., Lehky T., Bailey R.M., Chichester J.A., Calcedo R., Armao D. (2024). Intrathecal Gene Therapy for Giant Axonal Neuropathy. N. Engl. J. Med..

[88] Zielen S., Crawford T., Benatti L., Magnani M., Kieslich M., Ryan M., Meyts I., Gulati S., Borgohain R., Yadav R. (2024). Safety and efficacy of intra-erythrocyte dexamethasone sodium phosphate in children with ataxia telangiectasia (ATTeST): A multicentre, randomised, double-blind, placebo-controlled phase 3 trial. Lancet Neurol..

[89] Giacomini K.M., Huang S.-M., Tweedie D.J., Benet L.Z., Brouwer K.L.R., Chu X., Dahlin A., Evers R., Fischer V., Hillgren K.M. (2010). Membrane transporters in drug development. Nat. Rev. Drug Discov..

[90] Abbott N.J. (2013). Blood–brain barrier structure and function and the challenges for CNS drug delivery. J. Inherit. Metab. Dis..

[91] David S.H., Aniket S.W., Nathan B.R., Jimena G.P., Nina P.C., Victor F., Jeffrey A.W., Graeme F.W., Anthony J.K. (2016). Evolving Drug Delivery Strategies to Overcome the Blood Brain Barrier. Curr. Pharm. Des..

[92] Katz J.S., Slika H., Sattari S.A., Malla A.P., Xia Y., Antar A., Ran K., Tyler B. (2025). Overcoming the Blood-Brain Barrier for Drug Delivery to the Brain. ACS Omega.

[93] Yang H.-M. (2025). Overcoming the Blood–Brain Barrier: Advanced Strategies in Targeted Drug Delivery for Neurodegenerative Diseases. Pharmaceutics.

[94] Narsinh K.H., Perez E., Haddad A.F., Young J.S., Savastano L., Villanueva-Meyer J.E., Winkler E., de Groot J. (2024). Strategies to Improve Drug Delivery Across the Blood–Brain Barrier for Glioblastoma. Curr. Neurol. Neurosci. Rep..

[95] Liu S., Jin X., Ge Y., Dong J., Liu X., Pei X., Wang P., Wang B., Chang Y., Yu X.-A. (2025). Advances in brain-targeted delivery strategies and natural product-mediated enhancement of blood–brain barrier permeability. J. Nanobiotechnol..

[96] Majumder J., Taratula O., Minko T. (2019). Nanocarrier-based systems for targeted and site specific therapeutic delivery. Adv. Drug Deliv. Rev..

[97] Din F.U., Aman W., Ullah I., Qureshi O.S., Mustapha O., Shafique S., Zeb A. (2017). Effective use of nanocarriers as drug delivery systems for the treatment of selected tumors. Int. J. Nanomed..

[98] Ruman U., Fakurazi S., Masarudin M.J., Hussein M.Z. (2020). Nanocarrier-Based Therapeutics and Theranostics Drug Delivery Systems for Next Generation of Liver Cancer Nanodrug Modalities. Int. J. Nanomed..

[99] Aderibigbe B. (2018). In Situ-Based Gels for Nose to Brain Delivery for the Treatment of Neurological Diseases. Pharmaceutics.

[100] Husain A., Pandey N., Singh D., Ahmad F., Sharma R., Siddiqui M.H. (2023). Drug Discovery in Glioblastoma: Current Status and Future Perspectives. Biointerface Res. Appl. Chem..

[101] Persano F., Batasheva S., Fakhrullina G., Gigli G., Leporatti S., Fakhrullin R. (2021). Recent advances in the design of inorganic and nano-clay particles for the treatment of brain disorders. J. Mater. Chem. B.

[102] Bandopadhyay S., Manchanda S., Chandra A., Ali J., Deb P.K., Tekade R.K. (2020). Chapter 5—Overview of different carrier systems for advanced drug delivery. Drug Delivery Systems.

[103] Jena L., McErlean E., McCarthy H. (2020). Delivery across the blood-brain barrier: Nanomedicine for glioblastoma multiforme. Drug Deliv. Transl. Res..

[104] Zhou Y., Peng Z., Seven E.S., Leblanc R.M. (2018). Crossing the blood-brain barrier with nanoparticles. J. Control. Release.

[105] Crintea A., Dutu A.G., Sovrea A., Constantin A.-M., Samasca G., Masalar A.L., Ifju B., Linga E., Neamti L., Tranca R.A. (2022). Nanocarriers for Drug Delivery: An Overview with Emphasis on Vitamin D and K Transportation. Nanomaterials.

[106] Pandey A., Rath G., Chawala R., Goyal A.K. (2025). A comprehensive review on liraglutide and novel nanocarrier-based systems for the effective delivery of liraglutide. Naunyn-Schmiedeberg’s Arch. Pharmacol..

[107] Yang S., Aggarwal K., Jurczyszak J., Brown N., Sridhar S. (2024). Nanomedicine Therapies for Pediatric Diseases. Wiley Interdiscip. Rev. Nanomed. Nanobiotechnol.

[108] Račić A., Gatarić B., Topić Vučenović V., Stojmenovski A. (2025). Polysaccharide-Based Drug Delivery Systems in Pediatrics: Addressing Age-Specific Challenges and Therapeutic Applications. Polysaccharides.

[109] Teleanu D.M., Chircov C., Grumezescu A.M., Volceanov A., Teleanu R.I. (2018). Impact of Nanoparticles on Brain Health: An Up to Date Overview. J. Clin. Med..

[110] Chamundeeswari M., Jeslin J., Verma M.L. (2019). Nanocarriers for drug delivery applications. Environ. Chem. Lett..

[111] Khalbas A.H., Albayati T.M., Ali N.S., Salih I.K. (2024). Drug loading methods and kinetic release models using of mesoporous silica nanoparticles as a drug delivery system: A review. S. Afr. J. Chem. Eng..

[112] Wang N., Cheng X., Li N., Wang H., Chen H. (2019). Nanocarriers and Their Loading Strategies. Adv. Healthc. Mater..

[113] Petkar K.C., Chavhan S.S., Agatonovik-Kustrin S., Sawant K.K. (2011). Nanostructured materials in drug and gene delivery: A review of the state of the art. Crit. Rev. Ther. Drug Carr. Syst..

[114] Rafieezadeh D., Sabeti G., Khalaji A., Mohammadi H. (2025). Advances in nanotechnology for targeted drug delivery in neurodegenerative diseases. Am. J. Neurodegener. Dis..

[115] Ohta S., Kikuchi E., Ishijima A., Azuma T., Sakuma I., Ito T. (2020). Investigating the optimum size of nanoparticles for their delivery into the brain assisted by focused ultrasound-induced blood–brain barrier opening. Sci. Rep..

[116] Shilo M., Sharon A., Baranes K., Motiei M., Lellouche J.-P.M., Popovtzer R. (2015). The effect of nanoparticle size on the probability to cross the blood-brain barrier: An in-vitro endothelial cell model. J. Nanobiotechnol..

[117] Sun T., Zhang Y.S., Pang B., Hyun D.C., Yang M., Xia Y. (2014). Engineered Nanoparticles for Drug Delivery in Cancer Therapy. Angew. Chem. Int. Ed..

[118] Nsairat H., Khater D., Sayed U., Odeh F., Al Bawab A., Alshaer W. (2022). Liposomes: Structure, composition, types, and clinical applications. Heliyon.

[119] Juhairiyah F., De Lange E.C.M. (2021). Understanding Drug Delivery to the Brain Using Liposome-Based Strategies: Studies that Provide Mechanistic Insights Are Essential. AAPS J..

[120] Vieira D., Gamarra L. (2016). Getting into the brain: Liposome-based strategies for effective drug delivery across the blood-brain barrier. Int. J. Nanomed..

[121] Fulton M.D., Najahi-Missaoui W. (2023). Liposomes in Cancer Therapy: How Did We Start and Where Are We Now. Int. J. Mol. Sci..

[122] Raju R., Abuwatfa W.H., Pitt W.G., Husseini G.A. (2023). Liposomes for the Treatment of Brain Cancer—A Review. Pharmaceuticals.

[123] Zuberi A., Rehman U., Gupta G., Ghazwani M., Hani U., Kesharwani P. (2025). Smart liposomal systems for brain cancer: Technological innovations in drug delivery. Colloids Surf. B Biointerfaces.

[124] Song Y.H., De R., Lee K.T. (2023). Emerging strategies to fabricate polymeric nanocarriers for enhanced drug delivery across blood-brain barrier: An overview. Adv. Colloid Interface Sci..

[125] Zhang W., Mehta A., Tong Z., Esser L., Voelcker N.H. (2021). Development of Polymeric Nanoparticles for Blood-Brain Barrier Transfer-Strategies and Challenges. Adv. Sci..

[126] Caraway C.A., Gaitsch H., Wicks E.E., Kalluri A., Kunadi N., Tyler B.M. (2022). Polymeric Nanoparticles in Brain Cancer Therapy: A Review of Current Approaches. Polymers.

[127] Madani F., Esnaashari S.S., Webster T.J., Khosravani M., Adabi M. (2022). Polymeric nanoparticles for drug delivery in glioblastoma: State of the art and future perspectives. J. Control. Release.

[128] Su Y., Sun B., Gao X., Dong X., Fu L., Zhang Y., Li Z., Wang Y., Jiang H., Han B. (2020). Intranasal Delivery of Targeted Nanoparticles Loaded with miR-132 to Brain for the Treatment of Neurodegenerative Diseases. Front. Pharmacol..

[129] Chu L., Wang A., Ni L., Yan X., Song Y., Zhao M., Sun K., Mu H., Liu S., Wu Z. (2018). Nose-to-brain delivery of temozolomide-loaded PLGA nanoparticles functionalized with anti-EPHA3 for glioblastoma targeting. Drug Deliv..

[130] Fox L.J., Richardson R.M., Briscoe W.H. (2018). PAMAM dendrimer-cell membrane interactions. Adv. Colloid Interface Sci..

[131] Cook A.B., Perrier S. (2020). Branched and Dendritic Polymer Architectures: Functional Nanomaterials for Therapeutic Delivery. Adv. Funct. Mater..

[132] Sarode R.J., Mahajan H.S. (2024). Dendrimers for drug delivery: An overview of its classes, synthesis, and applications. J. Drug Deliv. Sci. Technol..

[133] Adnan Raza M., Firdous A., Gupta U., Patil U., Singh A., Ajazuddin (2025). Emerging Trends in Designing the Dendrimer-Based Drug Delivery for Antineoplastic Therapies. ACS Appl. Bio Mater..

[134] Yan X., Chen Q. (2024). Polyamidoamine Dendrimers: Brain-Targeted Drug Delivery Systems in Glioma Therapy. Polymers.

[135] Kaurav M., Ruhi S., Al-Goshae H.A., Jeppu A.K., Ramachandran D., Sahu R.K., Sarkar A.K., Khan J., Ashif Ikbal A.M. (2023). Dendrimer: An update on recent developments and future opportunities for the brain tumors diagnosis and treatment. Front. Pharmacol..

[136] Zhu Y., Liu C., Pang Z. (2019). Dendrimer-Based Drug Delivery Systems for Brain Targeting. Biomolecules.

[137] Ke W., Shao K., Huang R., Han L., Liu Y., Li J., Kuang Y., Ye L., Lou J., Jiang C. (2009). Gene delivery targeted to the brain using an Angiopep-conjugated polyethyleneglycol-modified polyamidoamine dendrimer. Biomaterials.

[138] Feliu N., Walter M.V., Montañez M.I., Kunzmann A., Hult A., Nyström A., Malkoch M., Fadeel B. (2012). Stability and biocompatibility of a library of polyester dendrimers in comparison to polyamidoamine dendrimers. Biomaterials.

[139] Pérez-Carrión M.D., Posadas I. (2023). Dendrimers in Neurodegenerative Diseases. Processes.

[140] Cagel M., Tesan F.C., Bernabeu E., Salgueiro M.J., Zubillaga M.B., Moretton M.A., Chiappetta D.A. (2017). Polymeric mixed micelles as nanomedicines: Achievements and perspectives. Eur. J. Pharm. Biopharm..

[141] Ghezzi M., Pescina S., Padula C., Santi P., Del Favero E., Cantù L., Nicoli S. (2021). Polymeric micelles in drug delivery: An insight of the techniques for their characterization and assessment in biorelevant conditions. J. Control. Release.

[142] Hwang D., Ramsey J.D., Kabanov A.V. (2020). Polymeric micelles for the delivery of poorly soluble drugs: From nanoformulation to clinical approval. Adv. Drug Deliv. Rev..

[143] Zhang Y., Huang Y., Li S. (2014). Polymeric Micelles: Nanocarriers for Cancer-Targeted Drug Delivery. AAPS PharmSciTech.

[144] Yousefpour Marzbali M., Yari Khosroushahi A. (2017). Polymeric micelles as mighty nanocarriers for cancer gene therapy: A review. Cancer Chemother. Pharmacol..

[145] Kaur J., Gulati M., Kapoor B., Jha N.K., Gupta P.K., Gupta G., Chellappan D.K., Devkota H.P., Prasher P., Ansari M.S. (2022). Advances in designing of polymeric micelles for biomedical application in brain related diseases. Chem.-Biol. Interact..

[146] Wu X.-Y., Liu J., Shen T., Wang J., Zhou J., Tang X., Xu L., Hong Z. (2014). Enhanced brain delivery of lamotrigine with Pluronic(®) P123-based nanocarrier. Int. J. Nanomed..

[147] Dasgupta A., May J.-N., Klinkenberg G., Besse H.C., Buhl E.M., Moeckel D., Mihyar R., Peña Q., Shalmani A.A., Hark C. (2025). Multidrug micelles and sonopermeation for chemotherapy co-delivery to brain tumors. J. Control. Release.

[148] Dykman L., Khlebtsov B., Khlebtsov N. (2025). Drug delivery using gold nanoparticles. Adv. Drug Deliv. Rev..

[149] Singh R., Mishra A.K., Bhushan B., Rawat H., Kumar V. (2024). A glance on gold nanoparticle: An emerging theranostic tool for oncology. J. Drug Deliv. Sci. Technol..

[150] Huang K., Ma H., Liu J., Huo S., Kumar A., Wei T., Zhang X., Jin S., Gan Y., Wang P.C. (2012). Size-Dependent Localization and Penetration of Ultrasmall Gold Nanoparticles in Cancer Cells, Multicellular Spheroids, and Tumors in Vivo. ACS Nano.

[151] Malik M.A., Hashmi A.A., Al-Bogami A.S., Wani M.Y. (2024). Harnessing the power of gold: Advancements in anticancer gold complexes and their functionalized nanoparticles. J. Mater. Chem. B.

[152] Panwar C., Phillips A., Sangal A., Gupta D. (2024). Synthesis of gold nanoparticles and their applications in cancer therapy. Biointerface Res. Appl. Chem..

[153] Venkataramani V., Tanev D.I., Strahle C., Studier-Fischer A., Fankhauser L., Kessler T., Körber C., Kardorff M., Ratliff M., Xie R. (2019). Glutamatergic synaptic input to glioma cells drives brain tumour progression. Nature.

[154] Beik J., Khateri M., Khosravi Z., Kamrava S.K., Kooranifar S., Ghaznavi H., Shakeri-Zadeh A. (2019). Gold nanoparticles in combinatorial cancer therapy strategies. Coord. Chem. Rev..

[155] Tu L., Luo Z., Wu Y.-L., Huo S., Liang X.-J. (2021). Gold-based nanomaterials for the treatment of brain cancer. Cancer Biol. Med..

[156] Cai Q., Li X., Xiong H., Fan H., Gao X., Vemireddy V., Margolis R., Li J., Ge X., Giannotta M. (2023). Optical blood-brain-tumor barrier modulation expands therapeutic options for glioblastoma treatment. Nat. Commun..

[157] Silveira G.d.B., Muller A.P., Machado-de-Ávila R.A., Silveira P.C.L. (2021). Advance in the use of gold nanoparticles in the treatment of neurodegenerative diseases: New perspectives. Neural Regen. Res..

[158] Puranik N., Yadav D., Song M. (2023). Advancements in the Application of Nanomedicine in Alzheimer’s Disease: A Therapeutic Perspective. Int. J. Mol. Sci..

[159] Zhao L., Lan T., Jiang G., Yan B. (2022). Protective effect of the gold nanoparticles green synthesized by Calendula officinalis L. extract on cerebral ischemia stroke-reperfusion injury in rats: A preclinical trial study. Inorg. Chem. Commun..

[160] Monfrini E., Baso G., Ronchi D., Meneri M., Gagliardi D., Quetti L., Verde F., Ticozzi N., Ratti A., Di Fonzo A. (2024). Unleashing the potential of mRNA therapeutics for inherited neurological diseases. Brain.

[161] Rabbani S.A., El-Tanani M., Sharma S., El-Tanani Y., Kumar R., Saini M., Yadav M., Khan M.A., Parvez S. (2025). RNA-Based Therapies for Neurodegenerative Diseases Targeting Pathogenic Proteins. Eur. J. Neurosci..

[162] Hosseinkhani H. (2024). Viral/Non-Viral Vectors in DNA/RNA Delivery Technology. Recent. Progress. Mater..

[163] High K.A., Roncarolo M.G. (2019). Gene Therapy. N. Engl. J. Med..

[164] Yin H., Kauffman K.J., Anderson D.G. (2017). Delivery technologies for genome editing. Nat. Rev. Drug Discov..

[165] Chen W., Yao S., Wan J., Tian Y., Huang L., Wang S., Akter F., Wu Y., Yao Y., Zhang X. (2021). BBB-crossing adeno-associated virus vector: An excellent gene delivery tool for CNS disease treatment. J. Control. Release.

[166] Ramamoorth M., Narvekar A. (2015). Non viral vectors in gene therapy- an overview. J. Clin. Diagn. Res. JCDR.

[167] Jindal D. (2025). Recent advances in gene therapy for neurological disorders: A comprehensive overview. Adesh Univ. J. Med. Sci. Res..

[168] Ozair A., Khan E., Bhat V., Faruqi A., Nanda A. (2022). Pediatric Brain Tumors: From Modern Classification System to Current Principles of Management.

[169] Louis D.N., Perry A., Wesseling P., Brat D.J., Cree I.A., Figarella-Branger D., Hawkins C., Ng H.K., Pfister S.M., Reifenberger G. (2021). The 2021 WHO Classification of Tumors of the Central Nervous System: A summary. Neuro Oncol..

[170] Kiviniemi A., Gardberg M., Ek P., Frantzén J., Bobacka J., Minn H. (2019). Gadolinium retention in gliomas and adjacent normal brain tissue: Association with tumor contrast enhancement and linear/macrocyclic agents. Neuroradiology.

[171] EMA (2017). EMA’s final opinion confirms restrictions on use of linear gadolinium agents in body scans. Med. Writ..

[172] FDA (2017). FDA Drug Safety Communication: FDA Warns that Gadolinium-Based Contrast Agents (GBCAs) are Retained in the Body; Requires New Class Warnings. https://www.sentinelinitiative.org/news-events/fda-safety-communications-labeling-changes/fda-warns-gadolinium-based-contrast-agents.

[173] Liu J., Wang T., Dong J., Lu Y. (2025). The blood–brain barriers: Novel nanocarriers for central nervous system diseases. J. Nanobiotechnol..

[174] Pourmasoumi P., Banihashemian S.A., Zamani F., Rasouli-Nia A., Mehrabani D., Karimi-Busheri F. (2024). Nanoparticle-Based Approaches in the Diagnosis and Treatment of Brain Tumors. J. Clin. Med..

[175] Xu J. (2025). Nanotechnology-Based Pharmacological Treatment for Brain Tumors. Highlights Sci. Eng. Technol..

[176] Kim J., Dey A., Malhotra A., Liu J., Ahn S.I., Sei Y.J., Kenney A.M., Macdonald T.J., Kim Y. (2020). Engineered biomimetic nanoparticle for dual targeting of the cancer stem-like cell population in sonic hedgehog medulloblastoma. Proc. Natl. Acad. Sci. USA.

[177] Choi J., Rui Y., Kim J., Gorelick N., Wilson D.R., Kozielski K., Mangraviti A., Sankey E., Brem H., Tyler B. (2020). Nonviral polymeric nanoparticles for gene therapy in pediatric CNS malignancies. Nanomed. Nanotechnol. Biol. Med..

[178] Bhowmik A., Chakravarti S., Ghosh A., Shaw R., Bhandary S., Bhattacharyya S., Sen P.C., Ghosh M.K. (2017). Anti-SSTR2 peptide based targeted delivery of potent PLGA encapsulated 3,3′-diindolylmethane nanoparticles through blood brain barrier prevents glioma progression. Oncotarget.

[179] Norouzi M., Yathindranath V., Thliveris J.A., Kopec B.M., Siahaan T.J., Miller D.W. (2020). Doxorubicin-loaded iron oxide nanoparticles for glioblastoma therapy: A combinational approach for enhanced delivery of nanoparticles. Sci. Rep..

[180] Chen H., Wen J. (2022). Iron oxide nanoparticles loaded with paclitaxel inhibits glioblastoma by enhancing autophagy-dependent ferroptosis pathway. Eur. J. Pharmacol..

[181] Poonaki E., Nickel A.-C., Shafiee Ardestani M., Rademacher L., Kaul M., Apartsin E., Meuth S.G., Gorji A., Janiak C., Kahlert U.D. (2022). CD133-Functionalized Gold Nanoparticles as a Carrier Platform for Telaglenastat (CB-839) against Tumor Stem Cells. Int. J. Mol. Sci..

[182] Kruse B., Dash B.S., Kostka K., Wolff N., Prymak O., Loza K., Gumbiowski N., Heggen M., Oliveira C.L.P., Chen J.-P. (2024). Doxorubicin-Loaded Ultrasmall Gold Nanoparticles (1.5 nm) for Brain Tumor Therapy and Assessment of Their Biodistribution. ACS Appl. Bio Mater..

[183] Liu C., Zhao Z., Gao H., Rostami I., You Q., Jia X., Wang C., Zhu L., Yang Y. (2019). Enhanced blood-brain-barrier penetrability and tumor-targeting efficiency by peptide-functionalized poly(amidoamine) dendrimer for the therapy of gliomas. Nanotheranostics.

[184] Uram Ł., Markowicz J., Misiorek M., Filipowicz-Rachwał A., Wołowiec S., Wałajtys-Rode E. (2020). Celecoxib substituted biotinylated poly(amidoamine) G3 dendrimer as potential treatment for temozolomide resistant glioma therapy and anti-nematode agent. Eur. J. Pharm. Sci..

[185] World Health Organization Epilepsy. https://apps.who.int/iris/bitstream/handle/10665/325293/9789241515931-eng.pdf?ua=1.

[186] Kowski A.B., Weissinger F., Gaus V., Fidzinski P., Losch F., Holtkamp M. (2016). Specific adverse effects of antiepileptic drugs—A true-to-life monotherapy study. Epilepsy Behav..

[187] Gernert M., Feja M. (2020). Bypassing the Blood–Brain Barrier: Direct Intracranial Drug Delivery in Epilepsies. Pharmaceutics.

[188] Thuraisingam S., Salim N., Azmi I.D.M., Kassim N.K., Basri H. (2023). Development of nanoemulsion containing Centella asiatica crude extract as a promising drug delivery system for epilepsy treatment. Biointerface Res. Appl. Chem..

[189] Sultana B., Panzini M.A., Veilleux Carpentier A., Comtois J., Rioux B., Gore G., Bauer P.R., Kwon C.S., Jetté N., Josephson C.B. (2021). Incidence and Prevalence of Drug-Resistant Epilepsy: A Systematic Review and Meta-analysis. Neurology.

[190] Besag F.M.C., Vasey M.J. (2021). Neurocognitive Effects of Antiseizure Medications in Children and Adolescents with Epilepsy. Pediatr. Drugs.

[191] Chen Z., Brodie M.J., Kwan P. (2020). What has been the impact of new drug treatments on epilepsy?. Curr. Opin. Neurol..

[192] Hughes S., Blumenthal M., Johnson A., Limjoco J., Lim S.Y. (2025). Pharmacokinetics and Pharmacodynamics of Levetiracetam in Neonatal Seizures: What We Still Need to Know. J. Pediatr. Pharmacol. Ther..

[193] Li S., Yi J., Tuo Y., Nie G., Wang J., Wang Y., Sun D., Liu Z. (2024). Population pharmacokinetics and dosing optimization of perampanel in children with epilepsy: A real-world study. Epilepsia.

[194] Wang W.-J., Li Y., Hu Y.-H., Wang J., Zhang Y.-Y., Fan L., Dai H.-R., Guo H.-L., Ding X.-S., Chen F. (2024). Population pharmacokinetics of valproic acid in children with epilepsy: Implications for dose tailoring when switching from oral syrup to sustained-release tablets. CPT Pharmacomet. Syst. Pharmacol..

[195] Bansal M., Dravid A., Aqrawe Z., Montgomery J., Wu Z., Svirskis D. (2020). Conducting polymer hydrogels for electrically responsive drug delivery. J. Control. Release.

[196] Pillay V., Tsai T.-S., Choonara Y.E., du Toit L.C., Kumar P., Modi G., Naidoo D., Tomar L.K., Tyagi C., Ndesendo V.M.K. (2014). A review of integrating electroactive polymers as responsive systems for specialized drug delivery applications. J. Biomed. Mater. Res. Part A.

[197] Wu D., Fei F., Zhang Q., Wang X., Gong Y., Chen X., Zheng Y., Tan B., Xu C., Xie H. (2022). Nanoengineered on-demand drug delivery system improves efficacy of pharmacotherapy for epilepsy. Sci. Adv..

[198] Dadfar S.M., Camozzi D., Darguzyte M., Roemhild K., Varvarà P., Metselaar J., Banala S., Straub M., Güvener N., Engelmann U. (2020). Size-isolation of superparamagnetic iron oxide nanoparticles improves MRI, MPI and hyperthermia performance. J. Nanobiotechnol..

[199] Xu H., Wang Y., Yu C., Han C., Cui H. (2024). Heparin-Modified Superparamagnetic Iron Oxide Nanoparticles Suppress Lithium Chloride/Pilocarpine-Induced Temporal Lobe Epilepsy in Rats through Attenuation of Inflammation and Oxidative Stress. ACS Chem. Neurosci..

[200] Rowley S., Patel M. (2013). Mitochondrial involvement and oxidative stress in temporal lobe epilepsy. Free Radic. Biol. Med..

[201] Waldbaum S., Patel M. (2010). Mitochondria, oxidative stress, and temporal lobe epilepsy. Epilepsy Res..

[202] Zhou Z., Li K., Chu Y., Li C., Zhang T., Liu P., Sun T., Jiang C. (2023). ROS-removing nano-medicine for navigating inflammatory microenvironment to enhance anti-epileptic therapy. Acta Pharm. Sin. B.

[203] Cevik O., Gidon D., Kizilel S. (2015). Visible-light-induced synthesis of pH-responsive composite hydrogels for controlled delivery of the anticonvulsant drug pregabalin. Acta Biomater..

[204] Rodrigues A.M., Santos L.E., Covolan L., Hamani C., de Almeida A.C. (2015). pH during non-synaptic epileptiform activity-computational simulations. Phys. Biol..

[205] Lu D., Ji Y., Sundaram P., Traub R.D., Guan Y., Zhou J., Li T., Zhe Sun P., Luan G., Okada Y. (2021). Alkaline brain pH shift in rodent lithium-pilocarpine model of epilepsy with chronic seizures. Brain Res..

[206] Trivedi R., Shende P. (2023). A stimuli-responsive nanocarrier for diagnosis of seizures and inhibition of glutaminase in epilepsy. Int. J. Pharm..

[207] Teleanu R.I., Niculescu A.-G., Vladacenco O.A., Roza E., Perjoc R.-S., Teleanu D.M. (2023). The State of the Art of Pediatric Multiple Sclerosis. Int. J. Mol. Sci..

[208] Leuzzi V., Nardecchia F., Pons R., Galosi S. (2021). Parkinsonism in children: Clinical classification and etiological spectrum. Park. Relat. Disord..

[209] Jan M.M. (2002). Clinical approach to children with suspected neurodegenerative disorders. Neurosci. J..

[210] Fusilli C., Migliore S., Mazza T., Consoli F., De Luca A., Barbagallo G., Ciammola A., Gatto E.M., Cesarini M., Etcheverry J.L. (2018). Biological and clinical manifestations of juvenile Huntington’s disease: A retrospective analysis. Lancet Neurol..

[211] Oosterloo M., Touze A., Byrne L.M., Achenbach J., Aksoy H., Coleman A., Lammert D., Nance M., Nopoulos P., Reilmann R. (2024). Clinical Review of Juvenile Huntington’s Disease. J. Huntington’s Dis..

[212] Cong W., Bai R., Li Y.-F., Wang L., Chen C. (2019). Selenium Nanoparticles as an Efficient Nanomedicine for the Therapy of Huntington’s Disease. ACS Appl. Mater. Interfaces.

[213] Teleanu D.M., Niculescu A.-G., Lungu I.I., Radu C.I., Vladâcenco O., Roza E., Costăchescu B., Grumezescu A.M., Teleanu R.I. (2022). An Overview of Oxidative Stress, Neuroinflammation, and Neurodegenerative Diseases. Int. J. Mol. Sci..

[214] Bhatt R., Singh D., Prakash A., Mishra N. (2015). Development, characterization and nasal delivery of rosmarinic acid-loaded solid lipid nanoparticles for the effective management of Huntington’s disease. Drug Deliv..

[215] Adhikari A., Mondal S., Das M., Biswas P., Pal U., Darbar S., Bhattacharya S.S., Pal D., Saha-Dasgupta T., Das A.K. (2021). Incorporation of a Biocompatible Nanozyme in Cellular Antioxidant Enzyme Cascade Reverses Huntington’s Like Disorder in Preclinical Model. Adv. Healthc. Mater..

[216] Cano A., Ettcheto M., Espina M., Auladell C., Folch J., Kühne B.A., Barenys M., Sánchez-López E., Souto E.B., García M.L. (2021). Epigallocatechin-3-Gallate PEGylated Poly(Lactic-Co-Glycolic) Acid Nanoparticles Mitigate Striatal Pathology and Motor Deficits in 3-Nitropropionic Acid Intoxicated Mice. Nanomedicine.

[217] Niemann N., Jankovic J. (2019). Juvenile parkinsonism: Differential diagnosis, genetics, and treatment. Park. Relat. Disord..

[218] Baskin J., Jeon J.E., Lewis S.J.G. (2021). Nanoparticles for drug delivery in Parkinson’s disease. J. Neurol..

[219] Mogharbel B.F., Cardoso M.A., Irioda A.C., Stricker P.E., Slompo R.C., Appel J.M., de Oliveira N.B., Perussolo M.C., Saçaki C.S., da Rosa N.N. (2022). Biodegradable Nanoparticles Loaded with Levodopa and Curcumin for Treatment of Parkinson’s Disease. Molecules.

[220] Kalčec N., Peranić N., Barbir R., Hall C.R., Smith T.A., Sani M.A., Frkanec R., Separovic F., Vinković Vrček I. (2022). Spectroscopic study of L-DOPA and dopamine binding on novel gold nanoparticles towards more efficient drug-delivery system for Parkinson’s disease. Spectrochim. Acta Part A Mol. Biomol. Spectrosc..

[221] Kalčec N., Peranić N., Mamić I., Beus M., Hall C.R., Smith T.A., Sani M.A., Turčić P., Separovic F., Vinković Vrček I. (2023). Selenium Nanoparticles as Potential Drug-Delivery Systems for the Treatment of Parkinson’s Disease. ACS Appl. Nano Mater..

[222] Elvidge K.L., Christodoulou J., Farrar M.A., Tilden D., Maack M., Valeri M., Ellis M., Smith N.J.C., Childhood Dementia Working Group (2023). The collective burden of childhood dementia: A scoping review. Brain.

[223] Djafar J.V., Johnson A.M., Elvidge K.L., Farrar M.A. (2023). Childhood Dementia: A Collective Clinical Approach to Advance Therapeutic Development and Care. Pediatr. Neurol..

[224] Nevin S.M., McGill B.C., Kelada L., Hilton G., Maack M., Elvidge K.L., Farrar M.A., Baynam G., Katz N.T., Donovan L. (2023). The psychosocial impact of childhood dementia on children and their parents: A systematic review. Orphanet J. Rare Dis..

[225] Ahmad S., Ahmad L., Adil M., Sharma R., Hasan N., Aqil M. (2025). Emerging nano-derived therapy for the treatment of dementia: A comprehensive review. Drug Deliv. Transl. Res..

[226] Shariare M.H., Rahman M., Lubna S.R., Roy R.S., Abedin J., Marzan A.L., Altamimi M.A., Ahamad S.R., Ahmad A., Alanazi F.K. (2020). Liposomal drug delivery of Aphanamixis polystachya leaf extracts and its neurobehavioral activity in mice model. Sci. Rep..

[227] Kaur A., Nigam K., Srivastava S., Tyagi A., Dang S. (2020). Memantine nanoemulsion: A new approach to treat Alzheimer’s disease. J. Microencapsul..

[228] Sood S., Jain K., Gowthamarajan K. (2013). P1–382: Curcumin-donepezil–loaded nanostructured lipid carriers for intranasal delivery in an Alzheimer’s disease model. Alzheimer’s Dement..

[229] Yusuf M., Khan M., Khan R.A., Ahmed B. (2013). Preparation, characterization, in vivo and biochemical evaluation of brain targeted Piperine solid lipid nanoparticles in an experimentally induced Alzheimer’s disease model. J. Drug Target..

[230] Zhang C., Wan X., Zheng X., Shao X., Liu Q., Zhang Q., Qian Y. (2014). Dual-functional nanoparticles targeting amyloid plaques in the brains of Alzheimer’s disease mice. Biomaterials.

[231] Gadhave D.G., Kokare C.R. (2019). Nanostructured lipid carriers engineered for intranasal delivery of teriflunomide in multiple sclerosis: Optimization and in vivo studies. Drug Dev. Ind. Pharm..

[232] Chountoulesi M., Demetzos C. (2020). Promising Nanotechnology Approaches in Treatment of Autoimmune Diseases of Central Nervous System. Brain Sci..

[233] Robinson A.P., Zhang J.Z., Titus H.E., Karl M., Merzliakov M., Dorfman A.R., Karlik S., Stewart M.G., Watt R.K., Facer B.D. (2020). Nanocatalytic activity of clean-surfaced, faceted nanocrystalline gold enhances remyelination in animal models of multiple sclerosis. Sci. Rep..

[234] Kumar P., Sharma G., Kumar R., Malik R., Singh B., Katare O.P., Raza K. (2017). Enhanced Brain Delivery of Dimethyl Fumarate Employing Tocopherol-Acetate-Based Nanolipidic Carriers: Evidence from Pharmacokinetic, Biodistribution, and Cellular Uptake Studies. ACS Chem. Neurosci..

[235] Lu L., Qi S., Chen Y., Luo H., Huang S., Yu X., Luo Q., Zhang Z. (2020). Targeted immunomodulation of inflammatory monocytes across the blood-brain barrier by curcumin-loaded nanoparticles delays the progression of experimental autoimmune encephalomyelitis. Biomaterials.

[236] Cho J.J., Stewart J.M., Drashansky T.T., Brusko M.A., Zuniga A.N., Lorentsen K.J., Keselowsky B.G., Avram D. (2017). An antigen-specific semi-therapeutic treatment with local delivery of tolerogenic factors through a dual-sized microparticle system blocks experimental autoimmune encephalomyelitis. Biomaterials.

[237] Saito E., Kuo R., Kramer K.R., Gohel N., Giles D.A., Moore B.B., Miller S.D., Shea L.D. (2019). Design of biodegradable nanoparticles to modulate phenotypes of antigen-presenting cells for antigen-specific treatment of autoimmune disease. Biomaterials.

[238] Osorio-Querejeta I., Carregal-Romero S., Ayerdi-Izquierdo A., Mäger I., Nash L.A., Wood M., Egimendia A., Betanzos M., Alberro A., Iparraguirre L. (2020). MiR-219a-5p Enriched Extracellular Vesicles Induce OPC Differentiation and EAE Improvement More Efficiently Than Liposomes and Polymeric Nanoparticles. Pharmaceutics.

[239] Yellepeddi V.K., Joseph A., Nance E. (2019). Pharmacokinetics of nanotechnology-based formulations in pediatric populations. Adv. Drug Deliv. Rev..

[240] Kapoor D.U., Garg R., Gaur M., Patel M.B., Minglani V.V., Prajapati B.G., Huanbutta K., Sangnim T., Sriamornsak P. (2024). Pediatric drug delivery challenges: Enhancing compliance through age-appropriate formulations and safety measures. J. Drug Deliv. Sci. Technol..

[241] Breitkreutz J., Boos J. (2007). Paediatric and geriatric drug delivery. Expert. Opin. Drug Deliv..

[242] Lim S.Y., Pettit R.S. (2019). Pharmacokinetic considerations in pediatric pharmacotherapy. Am. J. Health-Syst. Pharm. AJHP Off. J. Am. Soc. Health-Syst. Pharm..

[243] Niu S., Guo M., Yang H., Chang X., Shang M., Wu L., Ma Y., Xue Y. (2025). Ferroptosis effects and behavioral changes associated with high accumulation and low elimination percentage of silver in brain following a 28-day nasal instillation of silver nanoparticles in mice. Food Chem. Toxicol..

[244] Maher B.A. (2019). Airborne Magnetite- and Iron-Rich Pollution Nanoparticles: Potential Neurotoxicants and Environmental Risk Factors for Neurodegenerative Disease, Including Alzheimer’s Disease. J. Alzheimer’s Dis..

[245] Cabral H., Li J., Miyata K., Kataoka K. (2024). Controlling the biodistribution and clearance of nanomedicines. Nat. Rev. Bioeng..

[246] Powers C.M., Bale A.S., Kraft A.D., Makris S.L., Trecki J., Cowden J., Hotchkiss A., Gillespie P.A. (2013). Developmental neurotoxicity of engineered nanomaterials: Identifying research needs to support human health risk assessment. Toxicol. Sci. Off. J. Soc. Toxicol..

[247] Carpenter D., Gonzalez D., Retsch-Bogart G., Sleath B., Wilfond B. (2017). Methodological and Ethical Issues in Pediatric Medication Safety Research. Pediatrics.

[248] Turner M.A., Catapano M., Hirschfeld S., Giaquinto C. (2014). Paediatric drug development: The impact of evolving regulations. Adv. Drug Deliv. Rev..

[249] Rose K., Stötter H. (2006). ICH E 11: Clinical Investigation of Medicinal Products in the Paediatric Population.

[250] Sharland M., Zanichelli V., Ombajo L.A., Bazira J., Cappello B., Chitatanga R., Chuki P., Gandra S., Getahun H., Harbarth S. (2022). The WHO essential medicines list AWaRe book: From a list to a quality improvement system. Clin. Microbiol. Infect..

[251] Lagler F.B., Hirschfeld S., Kindblom J.M. (2021). Challenges in clinical trials for children and young people. Arch. Dis. Child..

[252] Mattsson H., Kindblom J.M., Norman E., Rane A., Naumburg E. (2020). Improved infrastructure and support needed for paediatric clinical trials in Sweden. Acta Paediatr..

[253] Mehrotra N., Bhattaram A., Earp J.C., Florian J., Krudys K., Lee J.E., Lee J.Y., Liu J., Mulugeta Y., Yu J. (2016). Role of Quantitative Clinical Pharmacology in Pediatric Approval and Labeling. Drug Metab. Dispos..

[254] Knowles J.K., Helbig I., Metcalf C.S., Lubbers L.S., Isom L.L., Demarest S., Goldberg E.M., George A.L., Lerche H., Weckhuysen S. (2022). Precision medicine for genetic epilepsy on the horizon: Recent advances, present challenges, and suggestions for continued progress. Epilepsia.

[255] Castellotti B., Ragona F., Freri E., Messina G., Magri S., Previtali R., Solazzi R., Franceschetti S., Taroni F., Canafoglia L. (2024). Next-generation sequencing in pediatric-onset epilepsies: Analysis with target panels and personalized therapeutic approach. Epilepsia Open.

[256] Gombolay G.Y., Gopalan N., Bernasconi A., Nabbout R., Megerian J.T., Siegel B., Hallman-Cooper J., Bhalla S., Gombolay M.C. (2023). Review of Machine Learning and Artificial Intelligence (ML/AI) for the Pediatric Neurologist. Pediatr. Neurol..

[257] Ren Q., Wu H., Zhang Y., Dai J., Chang Z., Nie J., Wang B., Fang Y. (2024). Nongenetic Precise Neuromodulation and Spatiotemporal Neuroprotection for Epilepsy Therapy via Rationally Designed Multifunctional Nanotransducer. ACS Nano.

[258] Nordli D.R., McLaren J.R., Araujo G., Gupta M., Nordli D.R., Galan F. (2024). Pediatric epilepsy syndromes with associated developmental impairment. Dev. Med. Child. Neurol..

